# Logic‐Gated HSV‐TK/GCV Suicide Gene Circuit for Triple‐Negative Breast Cancer

**DOI:** 10.1002/advs.202514749

**Published:** 2026-02-04

**Authors:** Shasha Tang, Yuan Fang, Lingli Jin, Dongyang Liu, Yicheng Liu, Ruijia Zheng, Liyun Yong, Xin Wu, Longliang Qiao, Meiyan Wang, Fengfeng Cai

**Affiliations:** ^1^ Department of Breast Surgery Tongji Hospital School of Medicine Tongji University Shanghai China; ^2^ Institute of Medical Technology Shanxi Medical University Taiyuan Shanxi Province China; ^3^ Shanghai 411 Hospital China RongTong Medical Healthcare Group Co. Ltd. 411 Hospital School of Medicine Shanghai University Shanghai China; ^4^ Chongqing Key Laboratory of Precision Optics Chongqing Institute of East China Normal University Chongqing China

**Keywords:** gene therapy, HSV‐TK/GCV system, off‐target toxicity, triple‐negative breast cancer

## Abstract

Triple‐negative breast cancer (TNBC) remains a major clinical challenge, owing to its molecular complexity, therapeutic resistance, and lack of specific druggable targets. The herpes simplex virus thymidine kinase/ganciclovir (HSV‐TK/GCV) suicide gene therapy system has shown promise in cancer treatment, but its clinical applicability is limited by off‐target cytotoxicity. Here, we developed a breast cancer‐specific suicide gene circuit (BRAS) that integrates the screened cancer‐specific promoters *RRM2* and *MAFK* with a microRNA specific to nontumor cells, utilizing the distinct molecular profiles of tumor and nontumor cells. This multi‐input logic gate circuit enables precise, specific expression of HSV‐TK in breast cancer cells with hardly expression in normal cell. We show that BRAS selectively induces apoptosis in patient‐derived TNBC cells while sparing normal cells. In two orthotopic breast cancer models, BRAS significantly suppressed tumor growth without affecting body weight or general health, underscoring its therapeutic potential. This approach intelligently combines molecular signals from both cancerous and healthy cells to precisely regulate therapeutic gene expression, making it a promising platform for the next‐generation cancer therapy.

## Graphical abstract

The BRAS comprises two modular genetic components driven by distinct tumor‐specific promoters and a failsafe layer with the NOT gate. This multi‐input logic gate circuit enables precise, specific expression of HSV‐TK in breast cancer cells with hardly expression in normal cell and effectively inhibits tumor growth in a triple‐negative breast cancer mouse model without off‐target toxicity.

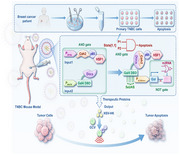

## 1 Introduction

Triple‐negative breast cancer (TNBC) is the most aggressive subtype of breast malignancy; it is defined by the absence of estrogen receptor, progesterone receptor, and HER2 expression and patients have a high relapse rate and poor prognosis. Standard treatment consists of surgery combined with neoadjuvant and adjuvant chemotherapy [1, 2, 3, 4]. However, surgery often fails to achieve complete tumor resection, and chemotherapy's nonselective cytotoxicity toward both malignant and normal cells can lead to severe side effects and the development of multidrug resistance. These limitations underscore an urgent need for innovative therapeutic approaches [5, 6, 7, 8].

So‐called suicide gene therapies represent a tumor‐selective strategy for eradicating malignant cells, which attracts a special attention because it allows self‐destruct enzyme gene into cancer cells, then give a harmless drug that only those cells can convert into a lethal poison, so the tumor kills itself while healthy tissue stays safe [9]. The herpes simplex virus thymidine kinase (HSV‐TK)/ganciclovir (GCV) system is one of the most widely studied suicide gene therapies [10, 11]. HSV‐TK phosphorylates the prodrug GCV into a toxic nucleotide analog that induces DNA chain termination, killing transduced tumor cells [12, 13, 14]. Despite the encouraging results of preclinical studies across multiple cancer types, including glioblastoma, lung, and breast cancer [13], the clinical efficacy of HSV‐TK/GCV remains limited because of insufficient tumor specificity driven by constitutive promoters.

Various natural and synthetic promoters have been employed to drive HSV‐TK expression seeking to enhance tumor selectivity, including promoters responsive to tumor‐associated conditions such as hypoxia, radiation, and oxidative stress. For example, the antioxidant response element (ARE)‐regulated HSV‐TK/GCV therapy provides improved selectivity and specificity of targeting cancer cells using the nuclear factor erythroid‐2 related factor 2 (Nrf2) overexpressed in human lung adenocarcinoma cells, which initiated ARE‐element to drive the expression of the HSV‐TK suicide gene in lung cancers [15]. Similarly, the hybrid promotor of STAT3 and NF‐κB was constructed to drive the expression of the HSV‐TK therapeutic protein in breast cancers, which can specifically target the STAT3/ NF‐κB activated tumor cells, subsequently suppressed tumor growth [9]. However, despite these innovations, suicide gene therapies have not yet achieved satisfactory clinical outcomes.

Genetic circuits have been developed to emulate digital logic through Boolean operations, such as AND, OR, and NOT, enabling cells to process complex input signals and respond with precise therapeutic actions [16, 17]. The synthetic gene circuits analyze tumor‐specific molecular patterns and implement targeted interventions with greater accuracy than conventional “always‐on” systems [18, 19, 20, 21]. For example, programmable oncolytic viruses and microRNA‐responsive circuits have been engineered to dynamically respond to tumor‐specific signals and evade immune suppression [22]. AND logic gates based on cancer‐specific promoter activity or protein levels have been applied in various cancer types [23], and an AAV‐compatible HCC cell classifier incorporating transcription factor and microRNA inputs have demonstrated tumor‐specific expression of HSV‐TK with minimal off‐target effects [24].

**FIGURE 1:**
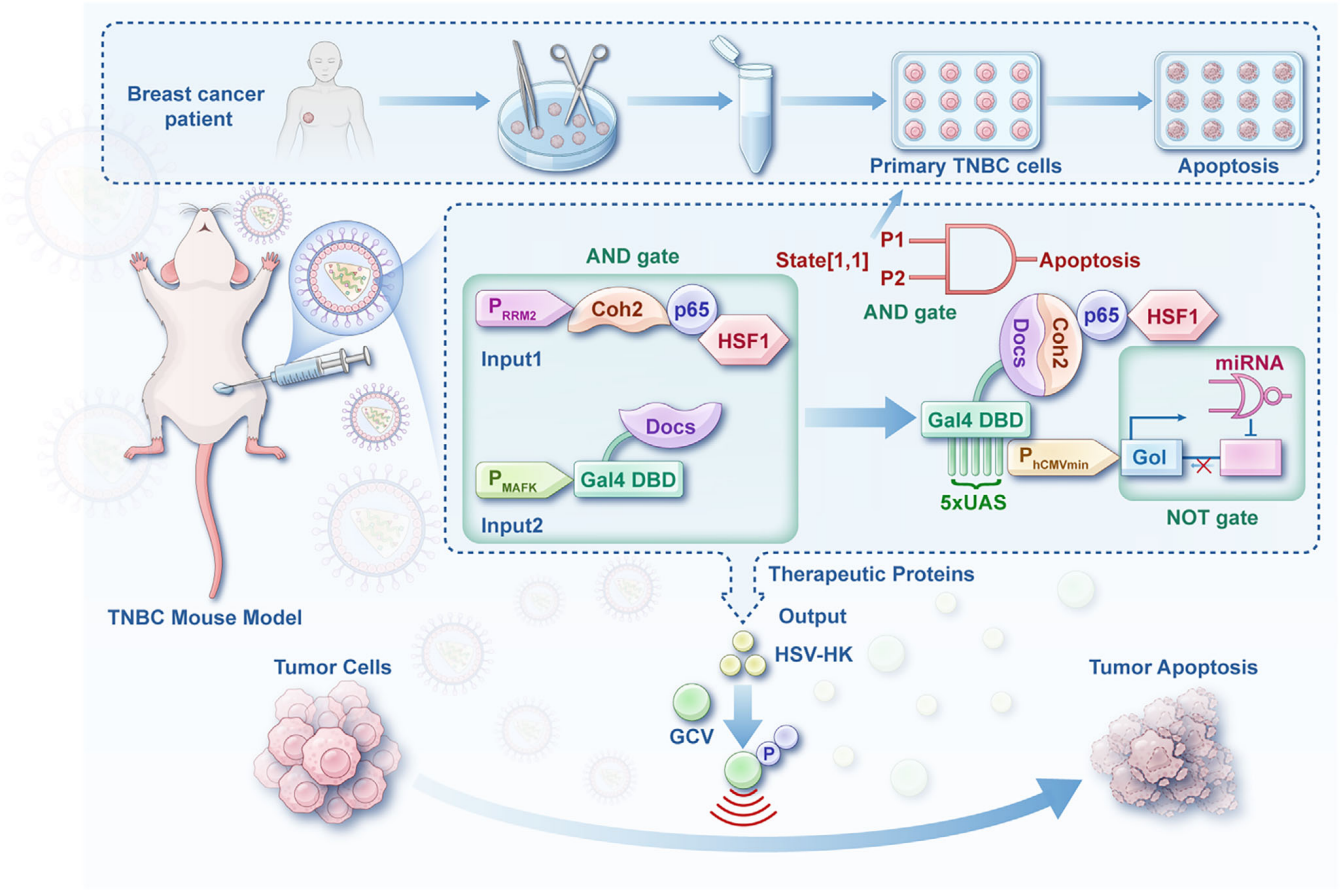
Schematic of the breast cancer‐specific suicide (BRAS) gene circuit for cancer therapy. The BRAS comprises two modular genetic components driven by distinct tumor‐specific promoters and a failsafe layer with the NOT gate for precise targeting of breast cancer cells. Input promoter 1 (P1, P_RRM2_) drives the expression of a hybrid transcriptional activator (Coh2‐p65‐HSF1), while input promoter 2 (P2, P_MAFK_) regulates a hybrid DNA‐binding module (Gal4 DBD‐DocS). In cells where both promoters are active, the Gal4 DBD binds the chimeric promoter (5×UAS‐P_hCMVmin_), and the high‐affinity interaction between the Coh2 and DocS domains enables transcriptional activation of the therapeutic effector. This logic‐gated AND circuit selectively initiates expression of the suicide gene with a NOT gate regulatory miRNA exclusively in breast cancer cells, sparing normal cells. This design effectively inhibits tumor growth in a triple‐negative breast cancer (TNBC) mouse model without off‐target toxicity.

In this study, we developed a breast cancer‐specific suicide gene circuit, termed breast cancer‐specific suicide (BRAS), based on a dual‐promoter AND gate and a failsafe layer with the NOT gate logic computation design. We selected two promoters (RRM2 and MAFK) that are highly expressed in breast cancer but not in normal cells, and incorporated microRNA‐205 (miR‐205) as an additional regulatory layer; miR‐205 is expressed abundantly in normal tissues but at very low in levels in TNBC cells. BRAS consists of two genetic modules: promoter 1 (P1, P_RRM2_) drives expression of a synthetic transcriptional activator (Coh2‐p65‐HSF1), while promoter 2 (P2, P_MAFK_) drives a hybrid DNA‐binding protein (Gal4 DBD‐Doc**S**). It is only in breast cancer cells (where both promoters are active) that these modules assemble, through a high‐affinity Coh2‐DocS interaction, to initiate transcription at a synthetic promoter (5×UAS‐P_hCMVmin_), leading to target gene expression in cancer cells. Meanwhile, microRNAs as a failsafe layer of NOT gate positioned right at the target locus shuts down target gene transcription in normal cells. We found the BRAS gene circuit enables tumor‐specific HSV‐TK expression in both immortalized and patient‐derived TNBC, but not in normal breast epithelial cells. Moreover, the BRAS circuit significantly inhibited tumor growth in the breast cancer models without inducing systemic toxicity or weight loss (Figure 1). Thus, our study provides a targeted suicide gene therapy system against breast cancers.

## 2 Results

### 2.1 Design and Construction of the BRAS Gene Circuit

**FIGURE 2:**
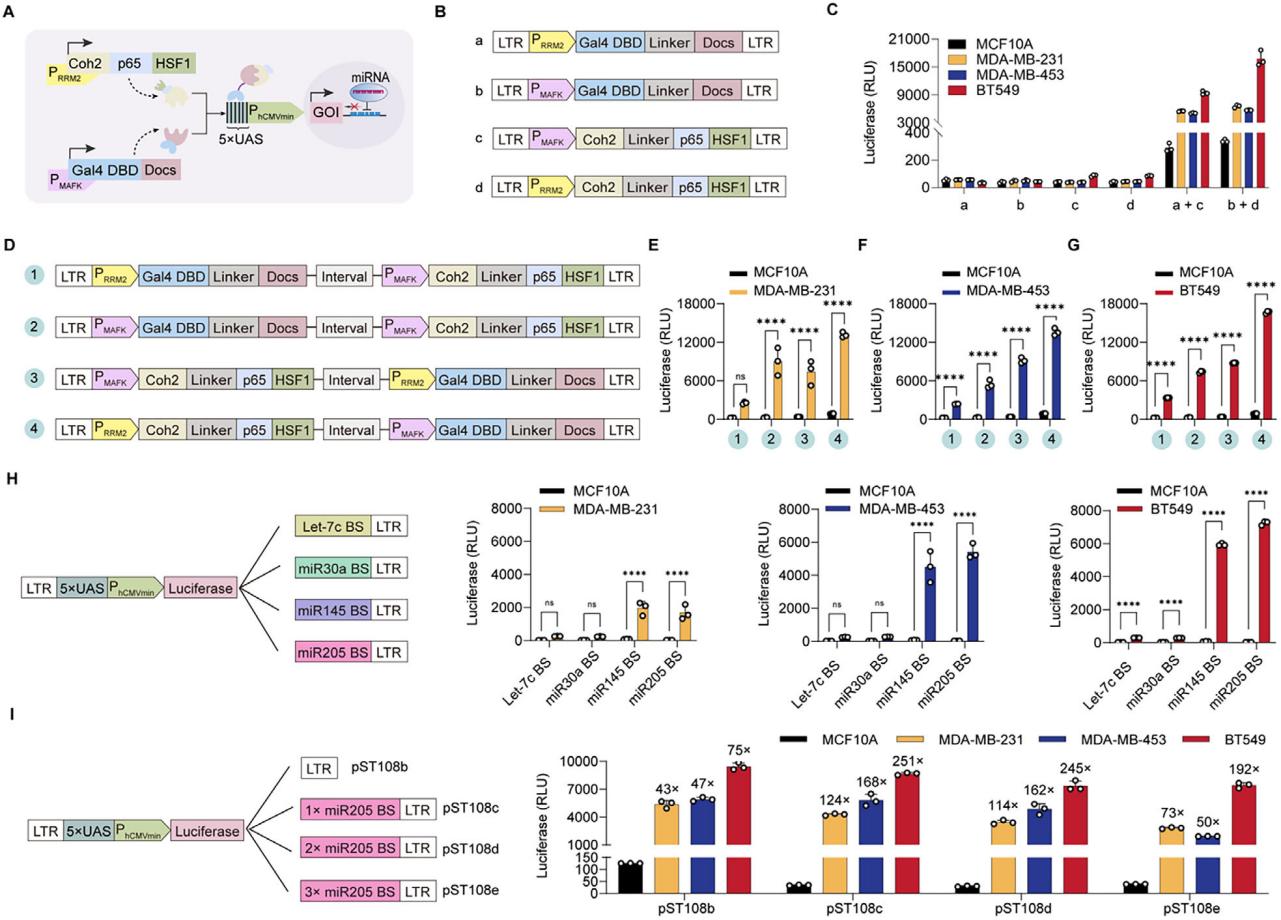
Design and optimization of the BRAS genetic circuit. (A) Schematic of the BRAS circuit components: a Coh2‐p65‐HSF1 hybrid transcriptional activator driven by P_RRM2_ (P1) and a Gal4 DBD‐DocS hybrid DNA‐binding protein driven by P_MAFK_ (P2). Upon coexpression, the interaction between Coh2 and Docs enables Gal4 DBD to bind the synthetic 5×UAS‐P_hCMVmin_ promoter, initiating output gene and miRNA expression. This transcript encoding miRNA inhibits the output gene transcript by targeting miRNA binding sites in the 3′‐untranslated region (3′‐UTR) of the output gene. (B) Configuration of AND gate genetic constructs used in BRAS circuit design. LTR: long terminal repeat. (C) Optimization of different AND gate configurations. Breast cancer cell lines (MDA‐MB‐231, MDA‐MB‐453, BT549) and normal epithelial cells (MCF10A; 3 × 10^4^ cells/well) were cotransduced with two combinations: lentiviral vectors encoding P_RRM2_ driven Gal4 DBD‐DocS and P_MAFK_ driven Coh2‐p65‐HSF1 (a + d), P_MAFK_ driven Gal4 DBD‐DocS and P_RRM2_ driven Coh2‐p65‐HSF1 (b + c), or P_RRM2_ driven Gal4 DBD‐DocS alone (a), or P_MAFK_ driven Gal4 DBD‐Docs alone (b), or P_RRM2_ driven Coh2‐p65‐HSF1 alone (c), or P_MAFK_ driven Coh2‐p65‐HSF1 alone (d), and a luciferase (Luci) reporter vector. Luciferase activity was measured 48 h post‐transduction. (D) Schematics of four concatenated construct variants combining Coh2‐p65‐HSF1 and Gal4 DBD‐DocS modules. (E–G) Performance optimization of these concatenated constructs. The breast cancer cell lines [MDA‐MB‐231 (E), MDA‐MB‐453 (F), BT549 (G)] and normal epithelial cells (MCF10A) were transduced with the four construct combinations and lentiviral Luci reporter vector. Luciferase expression was quantified after 48 h post‐transduction. (H) Evaluation of four miRNA binding site variants (*let‐7c‐5p*, *miR‐30a‐3p*, *miR‐145‐3p*, and *miR‐205‐5p*) for their regulatory effect on luciferase output. Cells were cotransduced with the lentiviral vectors encoding concatenated construct (P_RRM2_‐driven Coh2‐p65‐HSF1‐ P_MAFK_‐driven Gal4 DBD‐DocS), and Luci reporter alongside miRNA output vectors. Luciferase activity was assessed 48 h later. (I) Schematic of the bulged miR‐205 binding site (BS) design. Lentiviral vectors encoding the Luci reporter gene and varying copy numbers of miR‐205‐5p BS (1×, 2×, or 3×) were cotransduced with the BRAS circuit into breast cancer and MCF10A cells. Luciferase activity was quantified 48 h post‐transduction. Data in (C,E–I) are shown as mean ± SD (*n* = 3 independent experiments). *P*‐values in (C) were calculated via one‐way ANOVA with multiple comparisons; ns: not significant, ****P < 0.0001. *P*‐values in (E–I) were obtained via two‐tailed unpaired *t*‐test.

To generate a BRAS gene circuit, we constructed two distinct genetic modules, each regulated by a separate promoter. In this circuit, input promoter 1 (P1, P_RRM2_) drives expression of a hybrid transcriptional activator (Coh2‐p65‐HSF1), in which the transcriptional activator p65‐HSF1 was fused to the Coh2 domain derived from Clostridium thermocellum [25]. Input promoter 2 (P2, P_MAFK_) drives expression of a hybrid DNA‐binding protein (Gal4 DBD‐DocS), in which the yeast Gal4 DNA‐binding domain (Gal4 DBD) was fused to the Docs domain from the same bacterium [25]. When both promoters are active, the expressed Gal4 DBD domain is able to bind to a chimeric promoter (5×UAS‐P_hCMVmin_) [26], triggering expression of the therapeutic output gene through a high‐affinity interaction between the Coh2 and DocS domains (Figure 2A).

To identify optimal specific promoters driving gene of interest in breast cancer cells, we screened five candidates: [23, 27, 28, 29, 30] ribonucleotide reductase subunit M2 (*RRM2*), protein regulator of cytokinesis 1 (*PRC1*), β‐lactoglobulin (*Mamm*), mucin 1 (*MUC1*), and muscle aponeurosis fibrosarcoma oncogene homolog K (*MAFK*), using enhanced green fluorescent protein (EGFP) as a reporter. RRM2 and MAFK exhibited the strongest promoter activities in TNBC cell lines (MDA‐MB‐231, MDA‐MB‐453, and BT549) compared to normal breast epithelial MCF10A cells (Figure S1A). We further evaluated the specificity of RRM2 and MAFK promoters using a luciferase reporter in breast cancer subtypes (MCF7 [HR^+^], SKBR3 [HER2^+^], MDA‐MB‐231, MDA‐MB‐453, and BT549), as well as in other cancer cell types (HepG2 and HeLa) and in normal cells (RPE and MCF10A). And we found that there was high luciferase activity in breast cancer cells, particularly in TNBC lines, and a strong background in normal MCF10A cells, but not in HepG2, HeLa, or RPE (Figure S1B,C), indicating the specificity of RRM2 and MAFK promoters for breast cancer cells.

In order to reduce the background activity in normal cells, we engineered different BRAS promoter configuration and tested combinations of P_RRM2_ or P_MAFK_ driving Coh2‐p65‐HSF1 and Gal4 DBD‐DocS in TNBC cells (MDA‐MB‐231, MDA‐MB‐453, and BT549) and MCF10A cells (Figure 2B). The highest luciferase output was achieved when both modules were coactivated in TNBC cells, whereas negligible expression occurred when only one promoter was active (Figure 2C). Further, seeking a relatively simple system comprising few constructs to facilitate BRAS gene circuit delivery, we developed a single plasmid harboring both genetic modules (Figure 2D). We eventually obtained an optimized concatenated construct with P_RRM2_ driving Coh2‐p65‐HSF1 and P_MAFK_ driving Gal4 DBD‐DocS. To achieve long‐term gene expression and efficient packaging and delivery of the concatenated construct (about 5.5 kb), we selected lentiviral vectors delivery method for our BRAS circuit. We found that superior luciferase activity in all three TNBC cell lines through lentivirus infection. Specifically, this construct exhibited strongest luciferase activity in BT549 cells, while some leaky luciferase expression was maintained in normal MCF10A cells (Figure 2E–G).

To decrease leakage and improve systemic targeting precision, we incorporated NOT logic gates regulated by microRNAs (miRNAs) that are abundant in normal cells but suppressed in TNBC [24]. To implement the NOT gate, target sequences complementary to the miRNA inputs as miRNA sensor elements were incorporated into the 3′‐untranslated region (3′‐UTR) of the output gene. These miRNA sensor elements form an “incoherent feed‐forward” motif, which enhances repression of the output signal [31, 32]. Based on The Cancer Genome Atlas (TCGA) data [33], we selected five potential candidates including *let‐7c‐5p*, *miR‐30a‐3p*, *miR‐145‐3p*, and *miR‐205‐5p* as, which highly expressed in various healthy tissues, but not in breast cancer cells (Figure S2A–D), all of which were associated with better prognosis in breast cancer patients according to Kaplan‐Meier survival analysis (Figure S2E–H). qRT‐PCR analysis also showed that these miRNAs exhibited significantly lower expression levels in TNBC cell lines (MDA‐MB‐231, MDA‐MB‐453, and BT549) compared to MCF10A normal breast epithelial cells (Figure S3A–E).

To validate miRNA as a failsafe layer with the NOT gate suitable for output shutdown in normal cells in which the AND gate alone may not suffice, we inserted a single copy of these miRNA binding sequences into the 3′‐UTR of the output gene (Figure 2H). The BRAS constructs, including miRNA‐separated output reporter gene, were transduced into TNBC and MCF10A cells. Incorporation of *miR‐145‐3p* and *miR‐205‐5p* binding sites produced higher luciferase signal in breast cancer cells compared to normal cells. Notably, *miR‐205‐5p* induced the highest luciferase activity​​ among five candidate miRNAs assayed, ​​while only baseline activity in MCF10A control cells (Figure 2H).

Next, we tested different copy numbers of the *miR‐205‐5p* binding sequence (Figure 2I) and found that a single copy produced the highest fold‐change in luciferase expression between TNBC and normal cells (124‐fold in MDA‐MB‐231, 168‐fold in MDA‐MB‐453, and 251‐fold in BT549) and a little baseline activity (luciferase lever: about 35) in MCF10A control cells (Figure 2I). Based on this, we selected a single copy of the *miR‐205‐5p* binding sequence as the NOT gate input for enhancing the safety and selectivity of the BRAS circuit.

### 2.2 The In Vitro Effect of BRAS Circuit Mediated HSV‐TK/GCV

**FIGURE 3:**
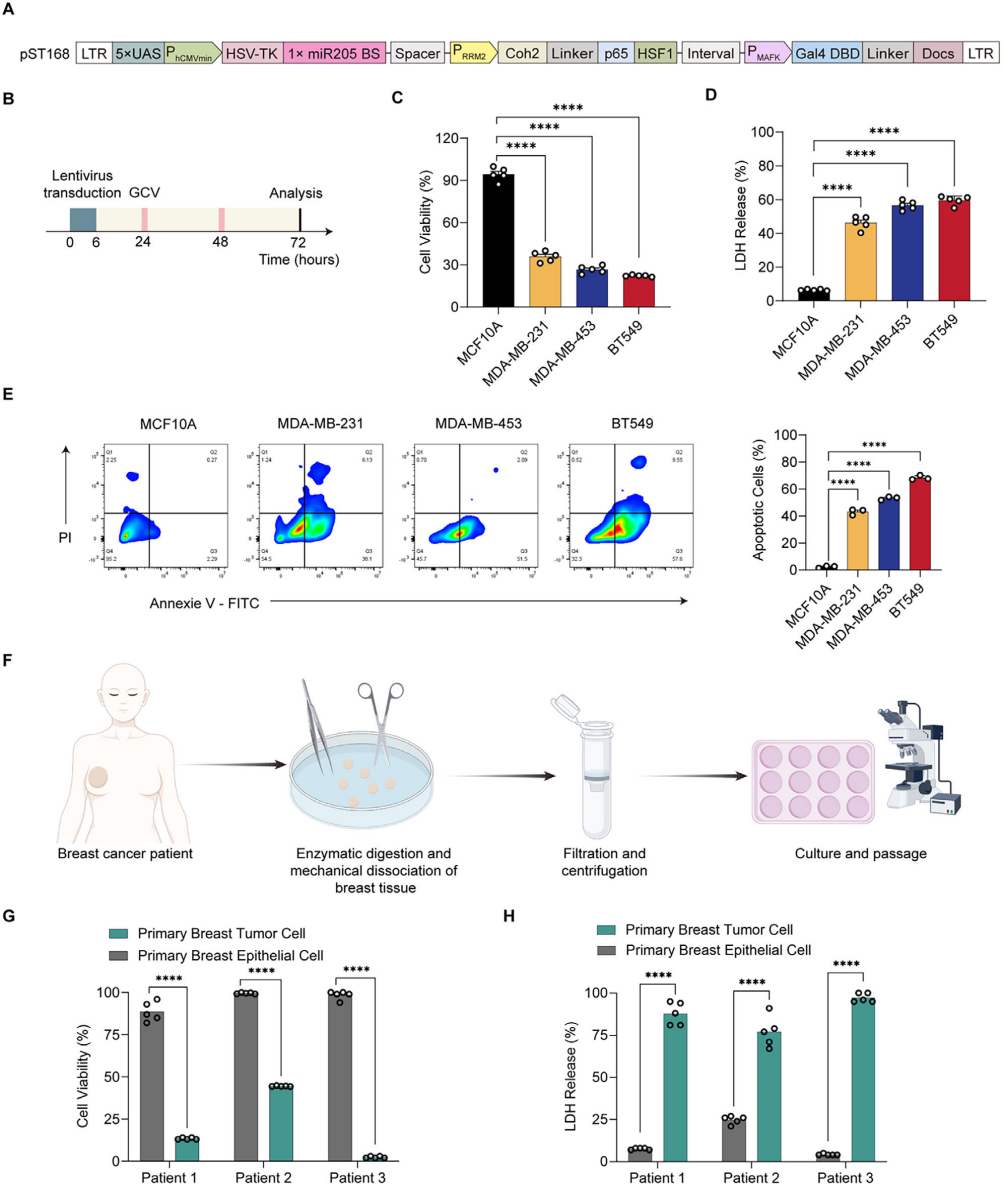
The BRAS circuit for breast cancer‐specific expression of therapeutic genes *HSV‐TK* in breast cancer cell lines and primary breast cancer cells. (A) Genetic configuration of the BRAS circuit encoding the therapeutic HSV‐TK gene and miR205. (B) Experimental timeline: breast cancer cells (3 × 10^4^ cells/well) were plated in 48‐well plates for 24 h, then cotransduced with lentiviral vectors encoding the BRAS circuit and HSV‐TK/mi205 output. GCV (3 mg/mL) was added 24 h post‐transduction, and cytotoxicity was evaluated after 48 h of GCV exposure. (C,D) Cell viability was measured in BRAS‐transduced MDA‐MB‐231, MDA‐MB‐453, and BT549 breast cancer lines and normal MCF10A cells after 72 h using the CCK‐8 assay (C) and LDH release assay (D). (E) Annexin V‐FITC/PI staining and flow cytometry analysis of apoptosis in BRAS‐transduced cells at 72 h. (F) Workflow for isolating primary TNBC patient‐derived cancer cells. (G,H) Viability assessment of patient‐derived TNBC cells and normal epithelial cells after BRAS‐mediated HSV‐TK/GCV treatment using CCK‐8 (G) and LDH release assays (H). Cells were cotransduced with the lentiviral vectors encoding the BRAS circuit and HSV‐TK/mi205 and treated with 3 mg/mL GCV for 72 h. Data in (C–E,G,H) are expressed as mean ± SD (*n* = 3 independent experiments). Statistical comparisons were made using one‐way ANOVA with multiple comparisons or two‐tailed unpaired *t*‐tests. ****P < 0.0001.

We next evaluated the antitumor efficacy of the BRAS circuit using HSV‐TK as the therapeutic output in combination with GCV. The HSV‐TK construct containing a single copy of the miR‐205‐5p binding site was packaged with the BRAS circuit into lentiviral vectors and transduced into six TNBC cell lines (MDA‐MB‐231, MDA‐MB‐453, BT549, EMT6, MDA‐MB‐468, and Hs 578T) and normal breast epithelial cells (MCF10A) (Figure 3A; Figure S4). Seventy‐two hours post‐transduction, cell viability was assessed using the CCK‐8 assay (Figure 3B). The BRAS circuit significantly suppressed cell viability in TNBC cells, reducing cell viability to 35.8%, 26.6%, 22.2%, 45.8%, 35.8%, and 29.4% in MDA‐MB‐231, MDA‐MB‐453, BT549, EMT6, MDA‐MB‐468, and Hs 578T (Figure S4A), respectively. In contrast, minimal cytotoxicity was observed in MCF10A cells (Figure 3C). Of note, strong EGFP expression and significant cell growth inhibition were observed in both TNBC cell lines (MDA‐MB‐231 and BT549) and normal cells (MCF10A) transduced with lentiviral vector encoding constitutively expressed HSV‐TK/GCV‐EGFP. In contrast, strong EGFP expression and cell death were only observed in TNBC cell lines (MDA‐MB‐231 and BT549) transduced with the BRAS constructs, while no EGFP fluorescence signal and minimal cytotoxicity were detected in normal cells (new Figure S8). These results indicated that the BRAS system markedly reduced off‐target toxicity.

To further assess cell death, we performed a lactate dehydrogenase (LDH) release assay to evaluate membrane integrity. Elevated LDH activity was detected in these TNBC cell lines transduced with the BRAS circuit compared to MCF10A, indicating increased cell lysis in TNBC cell lines (Figure 3D; Figure S4B). Cell apoptosis was also evaluated by Annexin V/propidium iodide (PI) staining and flow cytometry data showed approximately 40%–60% of TNBC cells transduced with the BRAS circuit underwent apoptosis, while apoptosis in MCF10A remained negligible (Figure 3E).

We further validated the therapeutic efficacy of the BRAS circuit in patient‐derived primary breast cancer cells. Following enzymatic digestion, filtration, and centrifugation, primary tumor and epithelial cells were isolated from clinical breast cancer patients (Figure 3F). The BRAS circuit exhibited significantly stronger cytotoxic effects in triple‐negative breast cancer cells compared to patient‐derived normal epithelial cells from TNBC patients (Figure 3G,H). In addition, we also assessed the bystander effect of BRAS circuit mediated HSV‐TK/GCV. CCK‐8 assay showed that the BT549 cells viability is 98.4%, 51.4%, 30.7%, 23.5%, 17.6%, and 13.4% in the percentage of 0%, 20%, 40%, 60%, 80%, and 100% BRAS‐transduced BT549 cells, indicating the bystander effect of HSV‐TK/GCV. By contrast, the MCF 10A cell proliferation was not affected with BRAS‐transduced BT549 cells (Figure S5). These results suggest that its selective therapeutic activity results from activation of HSV‐TK by both the AND gate and a second regulatory layer of the NOT gate logic.

### 2.3 Tumor‐Specific Inhibition by the BRAS Circuit With HSV‐TK Output in an Orthotopic Breast Cancer Mouse Model

**FIGURE 4:**
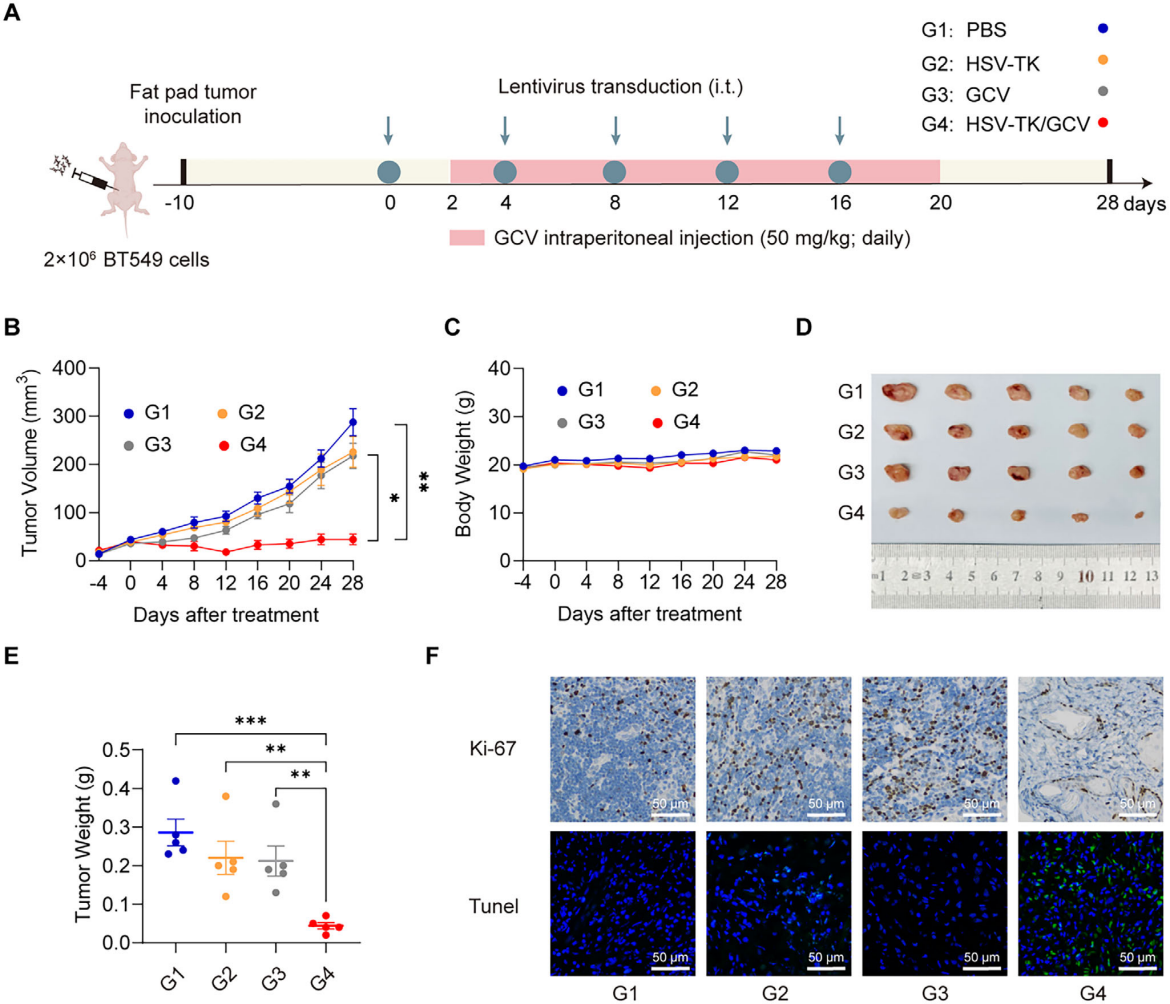
In vivo efficacy of the BRAS circuit‐mediated gene therapy prototype in an orthotopic TNBC mouse model. (A) Schematic illustration of the experimental timeline and procedure for evaluating the therapeutic efficacy of the BRAS circuit in an orthotopic BT549 TNBC mouse model. Female BALB/c nude mice (6 weeks old) were orthotopically injected with BT549 cells (2 × 10^6^ cells/mouse) into the mammary fat pad. Once tumors reached 30–50 mm^3^, mice were randomly assigned to four groups: (G1) PBS control, (G2) lentiviral vector encoding HSV‐TK, (G3) GCV alone, and (G4) lentiviral vector encoding BRAS circuit and HSV‐TK/miR205 output. Intratumoral injections of PBS, lentiviral vector encoding HSV‐TK/miRNA, or lentiviral vector encoding BRAS circuit and HSV‐TK/miR205 were administered on days 0, 4, 8, 12, and 16. Groups G3 and G4 also received daily intraperitoneal injections of GCV (50 mg/kg) from day 2 to day 20. (B) Tumor volumes were measured every four days until day 28 (*n* = 5). (C) Body weight was monitored throughout the experiment for all groups. (D,E) Representative images of excised tumors (D) and corresponding tumor weights (E). (F) Histological and immunohistochemical analyses of tumor sections collected at endpoint. Tumors were stained for Ki67 (brown) to assess proliferation and for TUNEL (green) to detect apoptosis, with DAPI (blue) counterstaining nuclei. Scale bar = 50 µm. Data in (B,C,E) are presented as mean ± SEM (*n* = 5 mice/group). Statistical significance was evaluated using two‐way ANOVA followed by Sidak's multiple comparisons test. *P < 0.05, **P < 0.01, ***P < 0.001.

Having established the in vitro efficacy of the BRAS circuit, we proceeded to evaluate its therapeutic potential in vivo using an orthotopic TNBC model. First, an orthotopic breast cancer mouse model was established by injecting into the mammary fat pad of immunodeficient mice. Ten days postinjection, once tumor volumes reached 50–100 mm^3^, mice were randomly divided into four groups and received the following treatments via intratumoral injection: (1) phosphate‐buffered saline (PBS, G1), (2) BRAS circuit vector with HSV‐TK output alone (G2), (3) GCV alone (G3), and (4) BRAS circuit vector with HSV‐TK output plus GCV (G4) (Figure 4A). Only G4 (HSV‐TK/GCV) group showed a marked inhibition of tumor growth and a significant reduction in tumor weight, whereas the control groups administered with PBS (G1), HSV‐TK alone (G2), or GCV alone (G3) showed exponential tumor burden increase over time (Figure 4B–E; Figure S6). The average tumor volume in the G4 group remained below 50 mm^3^, while tumors in all other groups exceeded 300 mm^3^ (Figure 4B). Importantly, no significant changes in body weight were observed across groups, suggesting minimal systemic toxicity during the treatment period (Figure 4C).

**FIGURE 5:**
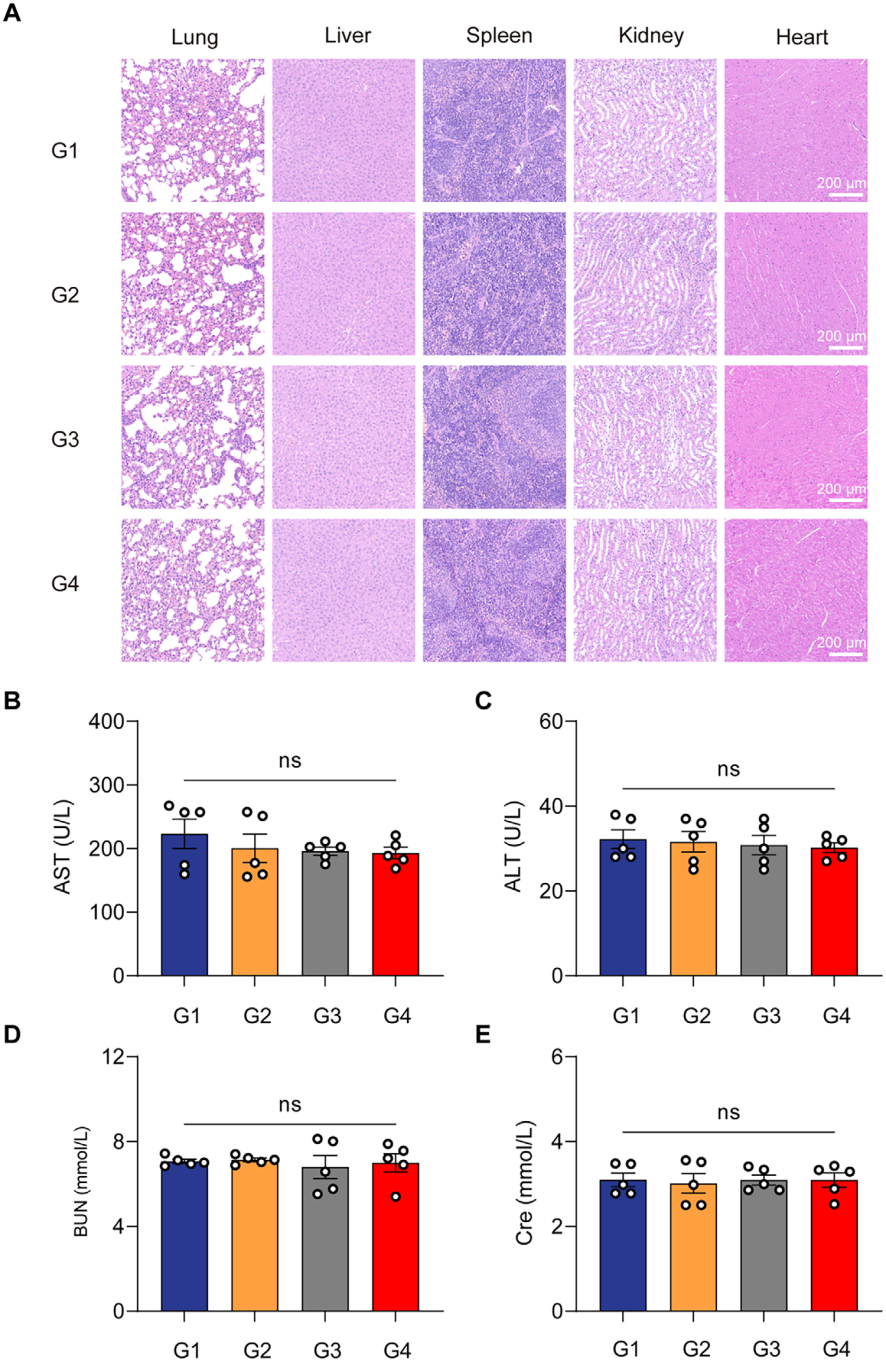
Biosafety of the BRAS circuit in an orthotopic TNBC mouse model. (A) Representative hematoxylin and eosin (H&E) staining images of major organs (lung, liver, spleen, kidney and heart) from BALB/c nude mice after 28 days of treatment. Scale bar = 200 µm. (B–E) Blood biochemical analysis to evaluate systemic toxicity: serum levels of (B) alanine aminotransferase (ALT), (C) aspartate aminotransferase (AST), (D) blood urea nitrogen (BUN), and (E) creatinine (CRE) were assessed using a BX‐3010 automatic biochemical analyzer (Sysmex). Data in (B–E) are presented as mean ± SEM (*n* = 5 mice/group). Statistical analyses were performed using two‐way ANOVA followed by Sidak's test. ns, not significant.

Tumor tissues from all groups were analyzed by hematoxylin and eosin (H&E) staining, Ki67 immunohistochemistry, and TUNEL assays. Tumors in the G4 group exhibited decreased Ki67 expression and increased TUNEL‐positive apoptotic cells, confirming reduced proliferation and enhanced cell apoptosis (Figure 4F). Histopathological examination of major organs (heart, liver, spleen, lungs, and kidneys) revealed no detectable abnormalities or tissue damage across all groups (Figure 5A). Additionally, blood serum analyses showed that liver enzymes including alanine aminotransferase (ALT) and aspartate aminotransferase (AST), and kidney function markers [blood urea nitrogen (BUN) and creatinine (CRE)] remained within normal physiological ranges (Figure 5B–E). In addition, we found that miR‐205‐5p was highly expression in normal tissues, whereas little miR‐205 was expressed in tumor tissues (Figure S7). These findings indicated a favorable systemic safety profile for the BRAS circuit.

**FIGURE 6:**
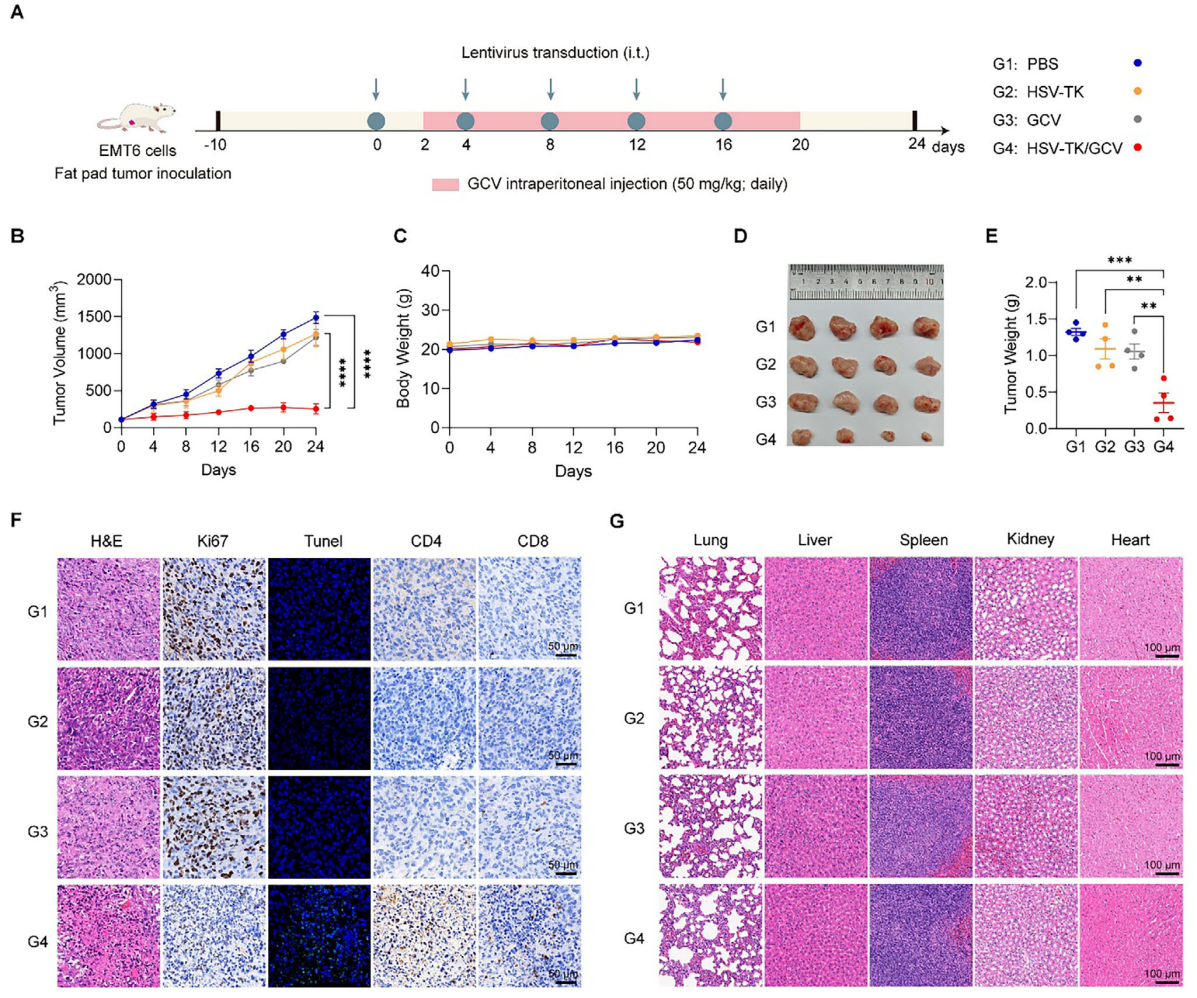
In vivo efficacy of the BRAS circuit‐mediated gene therapy prototype in immunocompetent Balb/c mice bearing EMT6 cells. (A) Schematic illustration of the experimental timeline and procedure for evaluating the therapeutic efficacy of the BRAS circuit in an orthotopic EMT6 mouse model. Female immunocompetent Balb/c mice (6–8 weeks old) were orthotopically injected with EMT6 cells (1 × 10^6^ cells/mouse) into the mammary fat pad. Once tumors reached 100–150 mm^3^, mice were randomly assigned to four groups: G1, phosphate‐buffered saline (PBS); G2, BRAS circuit vector with HSV‐TK output alone; G3, GCV; G4, BRAS circuit vector with HSV‐TK/miR205 output plus GCV. Intratumoral injections of PBS, lentiviral vector encoding HSV‐TK/miRNA, or lentiviral vector encoding BRAS circuit and HSV‐TK/miR205 were administered on days 0, 4, 8, 12, and 16. Groups G3 and G4 also received daily intraperitoneal injections of GCV (50 mg/kg) from day 2 to day 20. (B) Tumor volumes were measured every four days until day 24 (*n* = 4). (C) Body weight was monitored throughout the experiment for all groups. (D,E) Representative images of tumors (D) and corresponding tumor weights (E). (F) Representative images of HE, Ki67 (brown), TUNEL (green), CD4^+^ (brown), and CD8^+^ (brown) T cells staining in the tumors of different treatment groups. DAPI (blue) was stained for nuclei. Scale bar = 50 µm. (G) Representative hematoxylin and eosin (H&E) staining images of major organs (lung, liver, spleen, kidney and heart) from BALB/c immunocompetent mice after 24 days of treatment. Scale bar = 100 µm. Data in (B,C,E) are presented as mean ± SEM (*n* = 4 mice/group). Statistical significance was evaluated using two‐way ANOVA followed by Sidak's multiple comparisons test. **P < 0.01, ***P < 0.001, ****P < 0.0001.

To investigate bystander effects of the BRAS circuit with HSV‐TK output in vivo, an orthotopic breast cancer mouse model was established by injecting EMT6 cells into the mammary fat pad of immunocompetent Balb/c mice. The mice bearing EMT6 cells were randomly divided into four groups: G1, PBS; G2, BRAS circuit vector with HSV‐TK output alone; G3, GCV; G4, BRAS circuit vector with HSV‐TK output plus GCV. G4 exhibited a stronger tumor inhibitory effect against tumor growth compared to the PBS, HSV‐TK output alone, and GCV alone groups (Figure 6A–E). Tumor tissues were harvested for Ki67 and TUNEL assay, as well as H&E staining. The results demonstrated a marked reduction in proliferative capacity and a significant increase in apoptosis in the tumors of G4 mice compared to other control groups. Moreover, CD4^+^ and CD8^+^ T cells were high expression in tumor tissues from G4 group compared to control groups (Figure 6F). Additionally, there was no obvious histological changes were observed in the major organs including heart, liver, spleen, lung, and kidney in all groups (Figure 6G). These findings demonstrate that the BRAS circuit‐mediated bystander killing effect effectively inhibits tumor growth in the presence of GCV, with a favorable biosafety profile in vivo.

Collectively, beyond requiring the dual TNBC‐specific promoter AND gate, we also included a NOT gate in our BRAS circuit by introducing a miRNA binding site next to the gene‐of‐interest/therapeutic gene position, enabling an additional layer of control to prevent off‐target cytotoxicity. This study provides a promising therapeutic strategy for precision gene therapy in breast cancer.

## 3 Discussion

In the evolving landscape of gene therapy, synthetic biocomputation offers a transformative strategy by transferring the burden of cell‐type specificity from the delivery vector to the genetic circuitry itself [34, 35, 36, 37]. This shift enhances design flexibility and broadens the therapeutic scope to include diseases previously considered inaccessible or untargetable [38]. In this study, we constructed the BRAS gene circuit, which performed logical computation by integrating dual tumor‐specific promoter AND gate and the NOT gate as an additional failsafe layer of control that is at the level of transcription per se. This targeted design achieved breast cancer‐specific expression of HSV‐TK therapeutic transgene, as experimentally validated using six types of TNBC cell lines, patient‐derived tumor cells and matched normal epithelial counterparts.​

Although gene therapy holds tremendous promise for cancer treatment, translational targeting must meet the following requirements: high tumor selectivity and low off‐target toxicity [27, 39, 40]. Conventional transcriptional targeting strategies often rely on transcription factor or promoter that is also active in normal tissues, raising the risk of unintended cytotoxicity [38, 41, 42, 43, 44]. The tumor‐specific promoters variably expressed across cancers, offer a more selective mechanism for regulating therapeutic gene expression [45, 46, 47]. Our BRAS circuit provides a rational and effective strategy for precision targeting in TNBC, in which the target output is expressed at a high level only when the two promoters regulating both modules are mutually active, thereby improving specificity without compromising efficacy [22, 48, 49, 50, 51].

HSV‐TK‐based suicide gene therapy is a well‐established approach with demonstrated potential in a number of experimental and clinical settings [11, 15, 52, 53], but its clinical translation is hindered by off‐target toxicity due to leaky expression in nonmalignant tissues. To address this, our BRAS circuit tightly regulates *HSV‐TK* expression through a second regulatory layer of the failsafe NOT gate that exploits a uniquely weakly expressed miRNA in cancer cells. We showed that this circuit selectively induces apoptosis in TNBC cells with negligible effects on normal mammary epithelial cells. Moreover, in vivo application in two orthotopic mouse models elicited robust tumor inhibition with minimal systemic toxicity during the observed period, highlighting its translational potential for targeted suicide gene therapy in breast cancer. However, long‐term safety of this system needs to be evaluated before more generally applying this strategy.

Further, the BRAS circuit integrating dual tumor‐specific promoters and miRNA‐based NOT gate had dual‐layer control (transcriptional and post‐transcriptional control) of output expression, which reduced the risk of false activation compared to the single‐modality RNA‐based circuits. In the previously reported RNA‐based logic circuits, when a TF binds to target DNA, the temporal dynamics of the free TF concentration changes and leads to significant alterations in the behavior of genetic devices regulated by it [54]. This can cause effects ranging from changing the bias of bistable switches to going so far as to destroy sensitive temporal behaviors such as oscillations. Not only does this disrupt normal cellular processes, resource sharing also impacts the expression of supposedly independent components of the synthetic circuit, altering the dynamics of the system altogether [55, 56].

Lentivirus have been used the delivery tools for gene and cell therapy due to their unique biological features including a large packaging capacity, an efficient integration into the host cell genome, an intrinsic low immunogenicity in the general human population with a reduced capacity to induce inflammation and innate immune responses [49, [52, 57]. For example, the lentivirus‐based CAR‐T therapy was approved by the US FDA as CTL019 or tisagenlecleucel for refractory/relapsed Acute Lymphoblastic Leukemia (ALL) [58]. Recently, the lentiviral vector has been used for in vivo T‐cell engineering with a humanized anti‐B‐cell maturation antigen single‐domain‐antibody CAR [59]. In our study, the size of the BRAS circuit (about 5.5 kb) allowed packaging of it in lentiviral vectors, ensuring efficient delivery in vivo. Here, we have demonstrated that lentiviral vectors loaded with the BRAS circuit can efficiently transduce mouse tumors and express target genes.

Nonetheless, several challenges remain in optimizing the BRAS circuit for clinical application. Enhancing in vivo delivery efficiency is critical, particularly for systemic administration. Incorporating oncolytic viruses or engineered nonviral platforms, such as lipid nanoparticles and extracellular vesicles, may improve tumor tropism and payload delivery [60, 61, 62]. In addition, the circuit leakage in normal cells could be mitigated by experimentation which RRM2/MAFK promoters will be designed by artificial intelligence, especially deep learning techniques. This model is trained and tested by carrying out transcription rate measurements and TSS mapping on thousands of designed promoter sequences, followed by validation on the promoters characterized inside cells [63, 64]. Besides, cell heterogeneity may contribute to tumor escape by altering the logical result of the biocomputation. To minimize the risk of therapeutic escape, it may introduce a bystander‐killing output and/or multimodal effectors that integrate cytotoxic and immunomodulatory functions. Moreover, our preclinical validation employed two mouse models of breast cancer cells, further testing in immunocompetent systems—including syngeneic, humanized, and patient‐derived xenograft models—will be necessary to evaluate immune responses and account for tumor heterogeneity. These limitations would have to be addressed in follow‐up preclinical and clinical studies.

Finally, the modularity of our synthetic circuit opens avenues for combination therapy. The BRAS circuit can be readily adapted to encode immune‐modulating agents, such as nanobodies targeting immune checkpoints (e.g., anti‐CTLA‐4 or anti‐PD‐L1), thereby enabling simultaneous tumor cell killing and modulation of the tumor microenvironment. This circuit could be utilized to the expression of CAR‐T safety switches or ferroptosis inducers for cancer therapy. This sensor–computation–actuator framework, when extended to accommodate diverse inputs and outputs, holds promise for advancing next‐generation gene therapies that are both precise and safe.

## 4 Experimental Section

### 4.1 Ethical Statement

The study received approval from the Ethics Committee of Shanghai Tongji Hospital (No: SBKT‐2025‐219) and was conducted in accordance with Declaration of Helsinki. The human breast tumor specimens were provided by Tongji Hospital, with written informed consent obtained from the participants, and the study was approved by the Ethics Committee of Shanghai Tongji Hospital (No. 2025‐DW‐SB‐053). All animal experiments were conducted in accordance with the guidelines approved by the Animal Care and Use Committee at Shanghai Tongji Hospital and the Ministry of Science and Technology of the People's Republic of China on Animal Care Guidelines. All mice were euthanized after the termination of the experiments.

### 4.2 Construction of the BRAS Gene Circuit

The BRAS comprises two modular genetic components driven by distinct tumor‐specific promoters and a failsafe layer with the NOT gate for precise targeting of breast cancer cells. Input promoter 1 (P1, P_RRM2_) drives the expression of a hybrid transcriptional activator (Coh2‐p65‐HSF1), while input promoter 2 (P2, P_MAFK_) regulates a hybrid DNA‐binding module (Gal4 DBD‐DocS). In breast cancer cells where both promoters are active, the Gal4 DBD binds the chimeric promoter (5×UAS‐P_hCMVmin_), and the high‐affinity interaction between the Coh2 and DocS domains enables transcriptional expression of the downstream reporter or suicide gene. In normal cells, one fusion protein Coh2‐p65‐HSF1 do not dimerize with the other fusion protein Gal4 DBD‐Docs, thus failing to specifically bind to the chimeric promoter (5×UAS‐P_hCMVmin_) of the reporter or suicide gene plasmid and terminating the downstream gene expression.

### 4.3 Plasmids Construction

All plasmids used in this study (Table S1) were constructed via Gibson Assembly or restriction enzyme‐mediated cloning. The resulting constructs were sequence‐verified by Sanger sequencing (Genewiz).

### 4.4 Cell Culture

Human breast cancer cell lines including BT549 (Catalog no. TCHu93), SKBR3 (Catalog no. TCHu225), MCF7 (Catalog no. TCHu74), MDA‐MB‐453 (Catalog no. TCHu233), MDA‐MB‐231 (Catalog no. TCHu227), EMT6 (Catalog no. SCSP‐5499), MDA‐MB‐468 (Catalog no. TCHu136), Hs 578T (Catalog no. TCHu127), and human breast normal epithelial cell line (MCF10A, Catalog no. GNHu50) were obtained from the Chinese Academy of Sciences (Shanghai, China). BT549 and MDA‐MB‐468 were cultured in RPMI 1640 (Catalog no. 8122663, Gibco) with 10% fetal bovine serum (Catalog no. FBSSA500‐S, AusGeneX) and 1% penicillin/streptomycin mixture (Catalog no. ST488‐1/ST488‐2, Beyotime). MDA‐MB‐231, MCF7, SKBR3, MDA‐MB‐453, EMT6, Hs 578T, human embryonic kidney cell line (HEK293T, CRL‐11268, ATCC), human hepatocellular carcinoma cells (HepG2, ATCC), retinal pigment epithelial cells (RPE, ATCC), and human cervical adenocarcinoma cells (HeLa, CCL‐2, ATCC) were maintained in Dulbecco's modified Eagle's medium (DMEM, Catalog no. 12100061, Gibco) supplemented with 10% (v/v) fetal bovine serum, and 1% (v/v) penicillin/streptomycin solution. MCF10A cells were maintained in mammary epithelial cell medium supplemented with 1% penicillin–streptomycin solution, EGF, hormones, vitamins, and other necessary components (Catalog no. 7611, ScienCell). All cells were cultured at 37°C in a humidified incubator with 5% CO_2_ and were regularly tested for the absence of mycoplasma and bacterial contamination. The concentration and viability of the cell lines were assessed using a Countess II Automated cell counter (AMEP4746, Life Technologies). All cells have been confirmed to be free of mycoplasma contamination.

### 4.5 Cell Transfection

HEK‐293T cells were transfected using polyethyleneimine (PEI, MW 25,000) based protocol. Cells at ∼80% confluence were digested with 0.25% trypsin‐EDTA, resuspended, and seeded in 24‐well plates at 6 × 10^4^ cells/well 18 h before transfection. The plasmids and PEI (Catalog no. 24765, Polysciences; molecular weight 40,000, stock solution 1 µg/µL) were mixed at a mass ratio of 3:1 in 50 µL serum‐free medium, incubated for 15 min, and added to the cell culture wells. After 6 h of culture, the culture medium was refreshed to complete growth medium.

### 4.6 Lentiviral Packaging and Transduction

HEK‐293T cells were seeded at 7 × 10^6^ cells per 15 cm dish and cultured for 18 h before transfection. HEK‐293T cells were co‐transfected with lentiviral package plasmid (psPAX2, Catalog no. 12260, Addgene), a plasmid encoding for VSV‐G pseudotyping coat protein (pMD2G, Catalog no. 12259, Addgene), and target plasmid (37.5 µg total DNA) at a mass ratio of 2:1:2 using an optimized polyethyleneimine (PEI)‐based protocol with 1.5 mL of a 3:1 PEI:DNA mixture (w/w). Medium was replaced 6 h post‐transfection. Virus‐containing supernatants were harvested at 48 h, centrifuged at 2000 rpm for 10 min, filtered through a 0.45 µm syringe filter (Catalog no. 4654, Pall Corporation), and ultracentrifuged at 15,000 rpm for 2.5 h at 4°C using a Beckman Avanti J‐26 XPI centrifuge with a JA‐25.50 rotor (Beckman Coulter, Inc., CA, USA). Viral pellets were resuspended in 200 µL PBS, aliquoted, and stored at −80°C.

### 4.7 Lentiviral Titer Determination

MDA‐MB‐231 cells (3 × 10^4^ per well) were seeded in 48‐well plates and transduced with serial dilutions of concentrated lentiviral vector encoding EGFP (pLL3.7: LTR‐P_CMV_‐EGFP‐LTR) in the presence of 10 µg/mL polybrene. After 8 h, the medium was refreshed. Cells were harvested 48 h post‐transduction and transferred to 1.5 mL Eppendorf tubes, centrifuged at 350 × *g* for 5 min and resuspended in 0.5 mL of PBS. Samples were assessed by flow cytometry. The titer was calculated for those wells in which 5%–30% of the cells became transgene positive (the so‐called linear range). Higher transduction rates result in multiple integrations per cell and thus underestimation of the titer. The titer is calculated as follows: Titer (IU/mL) = (% GFP^+^ cells) × (number of plated cells) × (dilution factor)/(the volume of added supernatant). Multiplicity of Infection (MOI) is a ratio of infectious particles to the number of cells.

### 4.8 RNA Extraction and cDNA Synthesis

Total RNA was extracted from MCF10A, MDA‐MB‐231, MDA‐MB‐453, BT549 cells and mice tissues including heart, liver, spleen, lung, and kidney and tumor using the RNAiso Plus Kit (Catalog no. 9109, Takara Bio) according to the manufacturer's instructions. For first strand cDNA synthesis, 1000 ng total RNA was reverse‐transcribed using PrimeScript RT Reagent Kit with the genomic DNA Eraser (Catalog no. RR047, Takara Bio) and 1st Strand cDNA Synthesis Kit (Catalog no. R312‐01, Vazyme), following the manufacturers’ instructions.

### 4.9 Real‐Time PCR

Quantitative PCR (qPCR) analysis was performed on a real‐time PCR instrument (Roche, LightCycler 96, Switzerland), with U6 as internal control for normalizing miR‐205‐5p, miR‐145‐3p, Let‐7c‐5p, and miR‐30a‐3p expression. All primers were synthesized by Ruimian Biotechnology (China). The primers used for qPCR were showed in Table S2. The 2^−ΔΔCt^ method was employed to analyze the mRNA expression levels.

### 4.10 CCK‐8 Assay

Cell viability was assessed using the Cell Counting Kit‐8 (CCK‐8, Catalog no. C0037, Beyotime Biotechnology) following the manufacturer's protocol. MCF10A, MDA‐MB‐231, MDA‐MB‐453, and BT549 cells were seeded in 96‐well plates at 2000 cells/well and incubated overnight. Cells were transduced with lentiviral vector encoding the BRAS circuit: pST168 (LTR‐P_RRM2_‐Coh2‐linker‐p65‐HSF1‐interval‐P_MAFK_‐Gal4‐linker‐DocS‐LTR‐spacer‐LTR‐5×UAS‐P_hCMVmin_‐HSV‐TK‐miR205‐LTR, MOI = 3) in combination with GCV (Catalog no. sud‐gcv, InvivoGen) in the presence of 10 µg/mL polybrene (Catalog no. H9268; Sigma‐Aldrich) for 48 h. 10 µL of CCK‐8 solution was added to each well and incubated for 1 h at 37°C. Absorbance at 450 nm was recorded using the Synergy H1 reader (BioTek) with Gen5 software (version 2.04).

### 4.11 Lactate Dehydrogenase Release Assay

The cells were seeded in the 96‐well culture plate at 2000 cells/well and allowed to adhere overnight. Cells were transduced with lentiviral vector encoding the BRAS circuit pST168 (LTR‐P_RRM2_‐Coh2‐linker‐p65‐HSF1‐interval‐P_MAFK_‐Gal4‐linker‐DocS‐LTR‐spacer‐LTR‐5×UAS‐P_hCMVmin_‐HSV‐TK‐miR205‐LTR, MOI = 3) in combination with GCV in the presence of 10 µg/mL polybrene for 48 h. The supernatants of the cells were harvested. The quantification of LDH leakage was analyzed using a cytotoxicity LDH assay kit, according to the manufacturer's instructions. Finally, the absorbance at 490 nm was measured using a microplate reader (BioTek Instruments). Cell viability (%) was calculated as: (Sample absorbance − Blank absorbance)/(Control absorbance − Blank absorbance) × 100%.

### 4.12 Annexin V–FITC Apoptosis Detection

Apoptotic cells were identified using an Annexin V–FITC/PI Apoptosis Detection Kit (Catalog no. E606336, Sangon Biotech). MCF10A, MDA‐MB‐231, MDA‐MB‐453, and BT549 cells (3 × 10^4^) were transduced with lentiviral vector encoding the BRAS circuit pST168 (LTR‐P_RRM2_‐Coh2‐linker‐p65‐HSF1‐interval‐P_MAFK_‐Gal4‐linker‐DocS‐LTR‐spacer‐LTR‐5×UAS‐P_hCMVmin_‐HSV‐TK‐miR205‐LTR, MOI = 3) in combination with GCV in the presence of 10 µg/mL polybrene for 48 h before analysis. Cells were processed according to the manufacturer's instructions and analyzed on a BD LSRFortessa flow cytometer. FITC and PI fluorescence were detected using 488‐nm (530/30 nm filter, 505 nm long‐pass dichroic) and 561‐nm (610/20 nm filter, 595 nm long‐pass dichroic) channels, respectively. A gate was applied on forward scatter (FSC‐A) and side scatter (SSC‐A) to remove debris from cell populations. Data were processed using FlowJo V10 software.

### 4.13 Performance of the BRAS system

#### 4.13.1 Bystander Effect

BT549, MCF10A, and BRAS‐transduced BT549 cells were each digested with trypsin to prepare single‐cell suspensions. BRAS‐transduced BT549 cells were cocultured with untransduced MCF10A or BT549 cells in a 96‐well plate (1 × 10^4^ total cells per well), in which the percentage of BRAS‐transduced BT549 cells is 0%, 20%, 40%, 60%, 80%, and 100%. After 24 h, GCV (3 mg/mL) was added and cytotoxicity was evaluated after 48 h of GCV exposure by CCK‐8 assay. Absorbance at 450 nm was recorded using the Synergy H1 reader (BioTek) with Gen5 software (version 2.04).

#### 4.13.2 Off‐Target Toxicity and Tumor‐Killing Test

MDA‐MB‐231, BT549, and MCF10A cells were seeded in 96‐well plates at 2000 cells/well and incubated overnight. Cells were transduced with lentiviral vector encoding the BRAS circuit: pST171 (LTR‐5×UAS‐P_hCMVmin_‐HSV‐TK/GCV‐miR205‐P2A‐EGFP‐LTR, MOI = 3) and pST110 (LTR‐P_RRM2_‐Coh2‐Linker‐p65HSF1‐interval‐P_MAFK_‐Gal4‐Linker‐DocS‐LTR, MOI = 3) or the constitutively expressed pST170 (LTR‐P_hCMV_‐HSV‐TK/GCV‐P2A‐EGFP‐LTR, MOI = 3) in the presence of 10 µg/mL polybrene (Catalog no. H9268; Sigma‐Aldrich). After 24 h of incubation, the transduced cells were added with or without 3 mg/mL GCV (Catalog no. sud‐gcv, InvivoGen) for 48 h. For Off‐target toxicity experiment, the EGFP expression was observed and photographed by a fluorescence microscope (DMI8; Leica) equipped with an Olympus digital camera (Olympus DP71; Olympus). For tumor‐killing experiment, 10 µL of CCK‐8 solution was added to each well and incubated for 1 h at 37°C. Absorbance at 450 nm was recorded using the Synergy H1 reader (BioTek) with Gen5 software (version 2.04) in combination with or without 3 mg/mL GCV (Catalog no. sud‐gcv, InvivoGen).

### 4.14 Luciferase Assay

MCF10A, MDA‐MB‐231, MDA‐MB‐453, and BT549 cells (3 × 10^4^ per well) were transduced with lentiviral vector encoding the BRAS circuit including pST55 (LTR‐P_RRM2_‐Coh2‐linker‐p65‐HSF1‐LTR, MOI = 3), pST65 (LTR‐P_MAFK_‐Gal4‐linker‐DocS‐LTR, MOI = 3), and pST83 (LTR‐5×UAS‐P_hCMVmin_‐Luciferase‐LTR, MOI = 3) in the presence of 10 µg/mL polybrene for 48 h before analysis. Luciferase activity was measured using the Firefly Luciferase Reporter Gene Assay Kit (Catalog no. RG005, Beyotime Biotechnology). Briefly, the liquid culture was aspirated and 200 µL Cell Lysis Buffer was added to each well. After full lysis, each sample was centrifuged at 12,000 × *g* for 3 min and taken the supernatant for the assay. 100 µL of Firefly Luciferase Assay Reagent was added to each well of 100 µL samples and controls, and mixed well gently. Luminescence (RLU) was measured using a Synergy H1 hybrid multimode microplate reader (BioTek Instruments) with Gen5 software (version 2.04).

### 4.15 Isolation and Culture of Primary Human Breast Cancer Cells

Freshly excised human breast cancer tissue was immediately transferred to a biosafety cabinet for processing. For surface sterilization, the tissue was immersed in 75% ethanol for 15 min, followed by repeated rinses with PBS containing penicillin/streptomycin until the solution ran clear. Adipose tissue, blood vessels, and connective tissue were carefully removed using ophthalmic scissors. The residual tumor mass was finely minced and rinsed again. For enzymatic digestion, tissue fragments were incubated overnight at 4°C in a digestion buffer containing 0.1% collagenase types I (Catalog no. MB‐118‐0100, Rockland), collagenase types II (Catalog no. MB‐119‐0100, Rockland), and collagenase types IV (Catalog no. MB‐121‐0100, Rockland), along with 0.1% dispase (Catalog no. 40104ES80, YEASEN). The following day, the digested tissue was centrifuged, and the supernatant was discarded. The pellet was resuspended in 0.1% collagenase type II and incubated in a shaking 37°C water bath for 2–3 h. The resulting cell suspension was filtered through a 100‐mesh sieve, centrifuged at 300 × *g* for 5 min, and the cell pellet was cultured in complete DMEM medium at 37°C in a humidified incubator with 5% CO_2_.

### 4.16 Animals

All animals were approved by the Institutional Animal Care and Use Committee of Shanghai and conducted in accordance with the National Research Council Guide for Care and Use of Laboratory Animals. The experimental animals including 6‐week‐old female BALB/c nude mice and female immunocompetent BALB/c mice (6–8 weeks old) were reared in Tongji University Laboratory Animal Center.

### 4.17 In Vivo Evaluation of the BRAS Circuit in an Orthotopic TNBC Mouse Model

To assess the therapeutic efficacy of the BRAS circuit in vivo, an orthotopic TNBC model was established in female BALB/c nude mice (6 weeks old). BT549 cells (2 × 10^6^ cells/mouse) were orthotopically injected into the mammary fat pad. Once tumors reached a volume of 30–50 mm^3^, mice were randomly assigned to four treatment groups (*n* = 5 per group): (G1) PBS control, (G2) lentiviral vector encoding HSV‐TK alone, (G3) GCV prodrug alone, and (G4) lentiviral vector encoding the complete BRAS circuit with HSV‐TK output. Mice in groups G2 and G4 received intratumoral injections of the corresponding lentiviral vectors (1 × 10^8^ TU/mL) on day 0, 4, 8, 12, and 16. Mice in groups G3 and G4 were additionally administered GCV intraperitoneally at 50 mg/kg daily from day 2 to day 20. The tumor sizes of mice were measured using a digital caliper every four days and tumor growth was monitored by measuring tumor volume (length × width^2^/2) every four days until day 28. Body weight was recorded throughout the treatment period. At the endpoint, tumors were excised, photographed, weighed, and subjected to histopathological analysis, including H&E staining, TUNEL assay, immunohistochemistry (IHC), and blood biochemistry.

To investigate bystander effects of the BRAS circuit with HSV‐TK output in vivo, an orthotopic breast cancer mouse model was established in female immunocompetent Balb/c mice (6–8 weeks old). EMT6 cells (1 × 10^6^ cells/mouse) were orthotopically injected into the mammary fat pad. Once tumors reached a volume of 100–150 mm^3^, mice were randomly assigned to four treatment groups (*n* = 4 per group): G1, PBS; G2, BRAS circuit vector with HSV‐TK output alone; G3, GCV; G4, BRAS circuit vector with HSV‐TK output plus GCV. Mice in groups G2 and G4 received intratumoral injections of the corresponding lentiviral vectors (1 × 10^8^ TU/mL) on day 0, 4, 8, 12, and 16. Mice in groups G3 and G4 were additionally administered GCV intraperitoneally at 50 mg/kg daily from day 2 to day 20. Tumor growth was monitored by measuring tumor volume (length × width^2^/2), and body weight was recorded throughout the treatment period. At the endpoint, tumors were excised, photographed, weighed, and subjected to histopathological analysis, including H&E staining, TUNEL assay and IHC.

### 4.18 H&E Staining

Mice were euthanized via CO_2_ asphyxiation. Heart, liver, spleen, lung, kidney, and tumor tissues were collected, fixed in 4% paraformaldehyde (Catalog no. G1101, Servicebio) overnight at room temperature. The fixed samples were gently dehydrated through graded alcohol, cleared with xylene, and paraffin‐embedded. Sections (4 µm thick) were prepared using a Leica RM2235 rotary microtome, stained with H&E (Catalog no. G1005, Servicebio), and imaged using an Olympus BX53 upright microscope with a digital camera.

### 4.19 TUNEL Staining of Tumor Sections

Frozen tumor tissues were stained using a TUNEL Apoptosis Assay Kit (Catalog no. C1086, Beyotime Biotechnology) according to the manufacturer's protocol. In brief, tumor tissue sections were permeabilized with 0.3% Triton X‐100 in PBS for 5 min, followed by incubation with fluorescein‐labeled deoxyuridine triphosphate for 1 h. Slides were mounted with antifade medium, sealed, and imaged using a Leica DMI8 fluorescence microscope equipped with an Olympus DP71 digital camera. Apoptotic nuclei appeared green, while non‐apoptotic nuclei were stained blue.

### 4.20 Immumohistochemical Staining

After the paraffin sections of tumor tissue were dewaxed, antigen repair was performed on the paraffin sections with citrate buffer. After washing with 1× PBS, the sections were blocked by using 3% H_2_O_2_ for 5 min and then blocked for 15 min with 10% serum. After that, the primary antibodies including anti‐Ki67 (Catalog no. GB111141, 1:500, Servicebio), anti‐CD4 (Catalog no. GB15064, 1:200, Servicebio), and anti‐CD8 (Catalog no. GB15068, 1:400, Servicebio) were used to incubate the sections at 4°C overnight. The second antibody (Catalog no. GB23303, 1:200, Servicebio) was then used to incubate the sections at 37°C for 1 h. Afterward, the sections were stained by Diaminobenzidine (DAB, Catalog no. G1212, Servicebio) in the dark for 5 min, and redyed with hematoxylin (Catalog no. G1004, Servicebio) for 15 s. The sections were finally sealed with neutral gum and photgraphed using an Olympus BX53 upright microscope with a digital camera.

### 4.21 Hepatic and Kidney Function Analysis

Mice were euthanized, and their whole blood was collected. Plasma and serum samples were simultaneously analyzed for standard biochemical analytes. The parameters of hepatic function include alanine aminotransferase (ALT) and aspartate aminotransferase (AST). The parameters of kidney function, including creatinine (CRE) and blood urea nitrogen (BUN) were measured using an automatic biochemical analyzer BX‐3010 (Sysmex).

### 4.22 Statistical Analysis

All in vitro experiments were independently performed in triplicate, and data are presented as mean ± SD unless otherwise indicated in figure legends. For in vivo studies, treatment groups included 4‐5 randomly selected mice per group, and results are shown as mean ± SEM. No animals or data points were excluded from analysis. For comparisons between two groups, unpaired two‐tailed *t*‐tests were used. One‐way ANOVA followed by Dunnett's post hoc test was employed for multiple group comparisons with a single variable. Statistical analyses were performed using GraphPad Prism (version 9). Statistical significance was defined as *P < 0.05, **P < 0.01, ***P < 0.001, and ****P < 0.0001. Exact *n* and *P*‐values are reported in the relevant figures legends.

## Author Contributions

F.C. and M.W. conceived the project. F.C., M.W., S.T., Y.F., L.Q., and L.J. designed the experiments, analyzed the results, and wrote the manuscript. S.T., Y.F., L.Q., L.J., D.L., Y.L., R.Z., L.Y., and X.W. performed the experimental work. S.T., Y.F., L.J., M.W., L.Q., and F.C. designed, analyzed, and interpreted the experiments. All authors edited and approved the manuscript.

## Funding

This work was supported by the National Natural Science Foundation of China (No. 32171414), the Natural Science Foundation of Shanghai (No. 23ZR1419500), and the Nature Science Foundation of Chongqing, China (No. CSTB2022NSCQ‐MSX0461) to M.W., the Young Scientists Fund of the National Natural Science Foundation of China (No. 3250120102), the Postdoctoral Fellowship Program and China Postdoctoral Science Foundation (Nos. BX20250121 and 2025M772716) to L.Q. This work was supported by grants from the Sixth Cycle Key Discipline Funding from Tongji Hospital, School of Medicine, Tongji University (No. ZDTS24‐RX) and by grants from the Tongji Hospital, School of Medicine, Tongji University (Nos. GJPY2337 and GJPY2402).

## Conflicts of Interest

The authors declare no conflict of interest.

## Supporting information




**Supporting File**: advs74211‐sup‐0001‐SuppMat.docx.

## Data Availability

The data that support the findings of this study are available from the corresponding author upon reasonable request.
