# Supplementary material for: Logic‐Gated HSV‐TK/GCV Suicide Gene Circuit for Triple‐Negative Breast Cancer
Source: Adv Sci (Weinh). 2026 Feb 4;13(20):e14749. doi: 10.1002/advs.202514749 (PMC13067838; doi:10.1002/advs.202514749)
Supplement: Supplementary file 1 — Supporting File: advs74211‐sup‐0001‐SuppMat.docx. [file ADVS-13-e14749-s001.docx]

**Supporting Information**

**L****ogic-Gated HSV-TK/GCV Suicide Gene Circuit for Triple-Negative Breast Cancer**

*Shasha Tang^#^, Yuan Fang^#^, Lingli Jin^#^, Dongyang Liu, Yicheng Liu, Ruijia Zheng, Liyun Yong, Xin Wu, Longliang Qiao^*^, Meiyan Wang^*^, Fengfeng Cai^*^*

S.T., Y.F., L.J., D.L., Y.L., R.Z., L.Y., L.Q., F.C.,

Department of Breast Surgery, Tongji Hospital, School of Medicine, Tongji University, Shanghai 200065, China

E-mail: [qiaolongliang1@126.com](mailto:qiaolongliang1@126.com), [caifengfeng@tongji.edu.cn](mailto:caifengfeng@tongji.edu.cn)

X.W.,

Institute of Medical Technology, Shanxi Medical University, Taiyuan, Shanxi Province 030001, China

M.W.,

Shanghai 411 Hospital, China RongTong Medical Healthcare Group Co.Ltd., 411 Hospital, School of Medicine, Shanghai University, Shanghai 200444, China

Chongqing Key Laboratory of Precision Optics, Chongqing Institute of East China Normal University, Chongqing 401120, China

E-mail: [wangmy@shu.edu.cn](mailto:wangmy@shu.edu.cn)

**
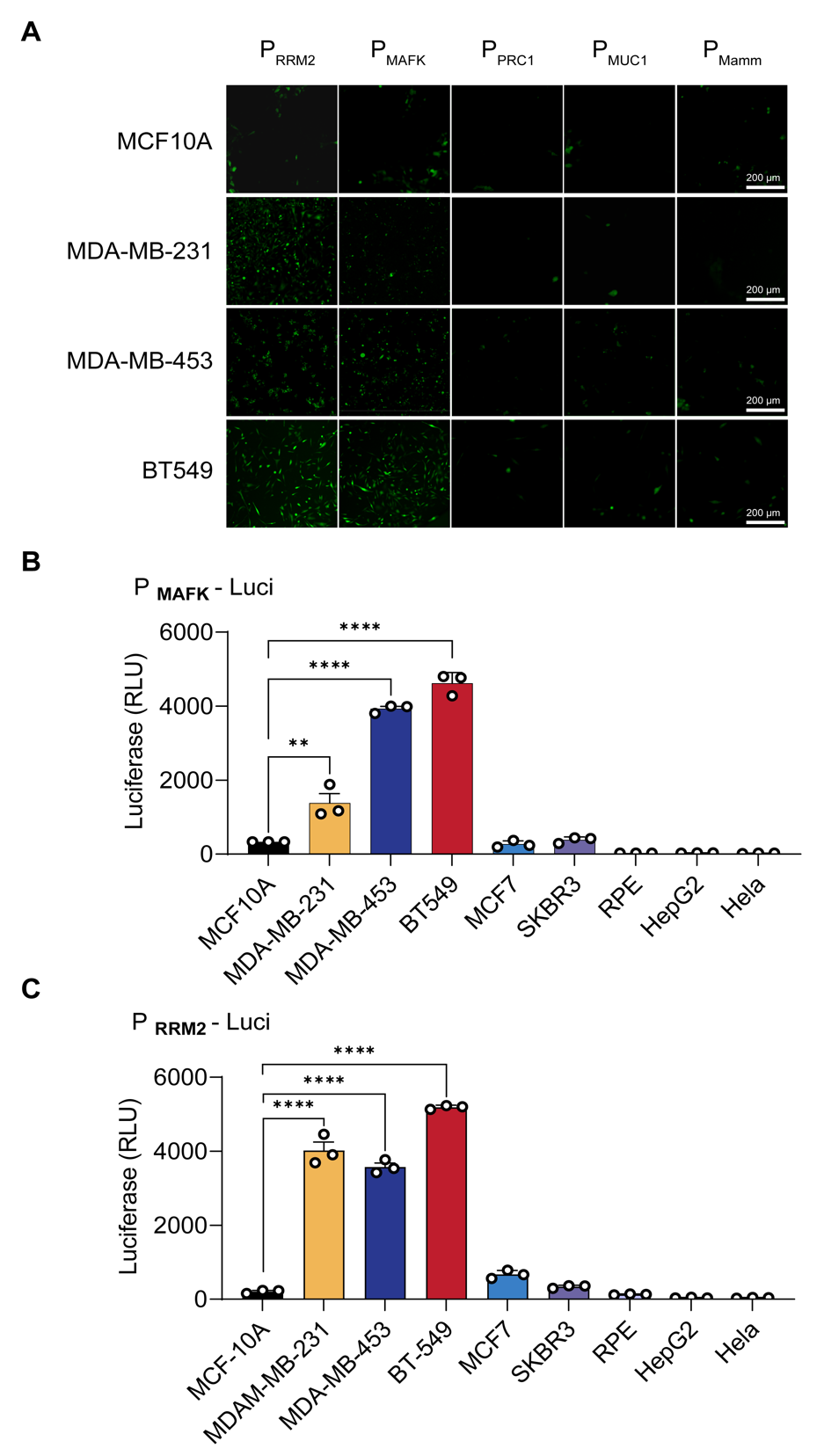
**

**Figure S1. Screening and validation of Breast Cancer-Specific Promoters.** (**A**) Promoters *RRM2*, *MAFK*, *PRC1*, *MUC1*, and *Mamm* were used to drive EGFP expression. MCF10A, MDA-MB-231, MDA-MB-453, and BT549 cells were infected, and EGFP fluorescence was visualized. Scale bar = 200 μm. (**B-C**) Luciferase activity driven by RRM2 and MAFK promoters was assessed across various cell lines: RPE (retinal pigment epithelial), HepG2 (liver cancer), HeLa (cervical cancer), MCF7 (HR⁺ breast cancer), SKBR3 (HER2⁺ breast cancer), BT549, MDA-MB-231, MDA-MB-453 (TNBC), and MCF10A (normal breast epithelial).


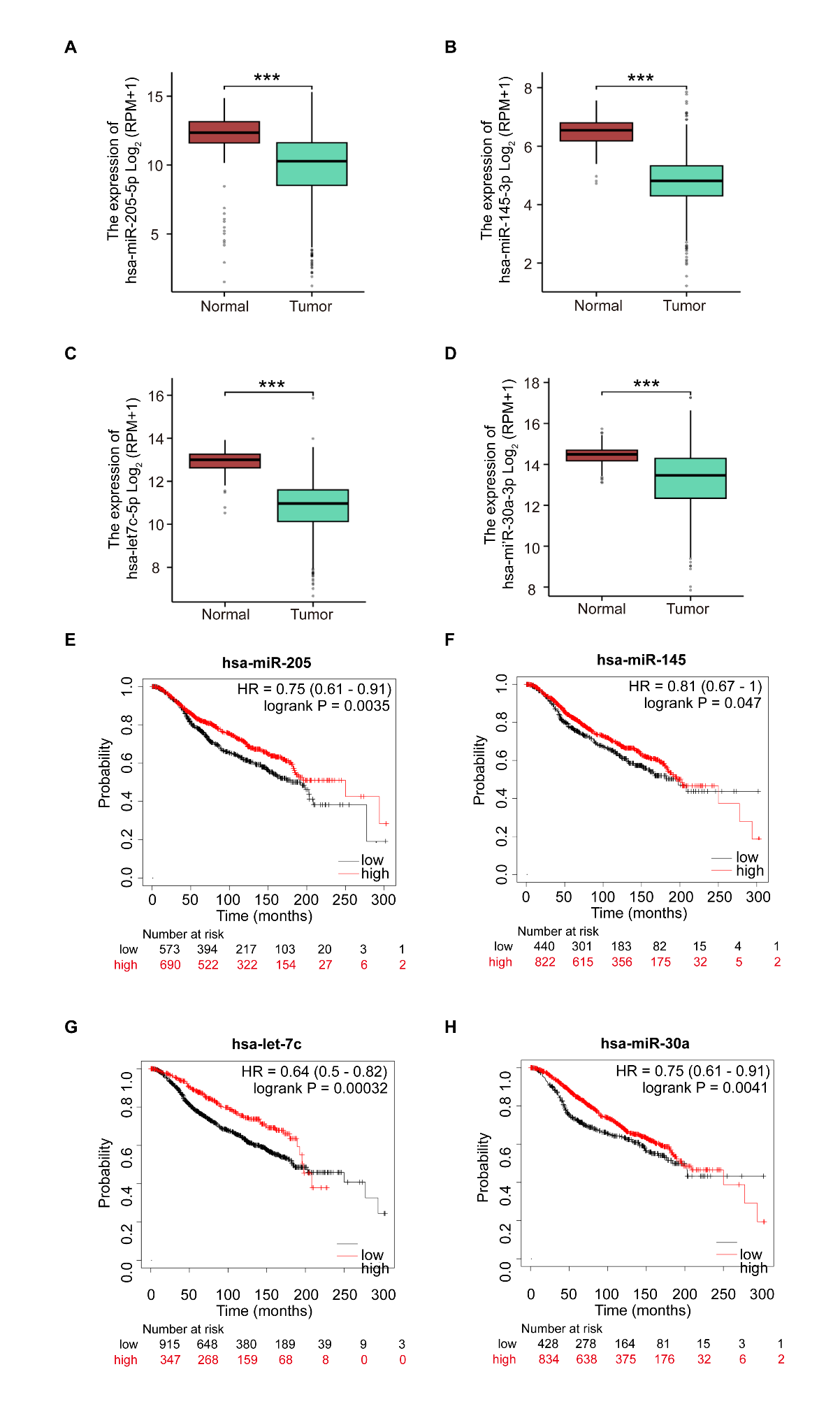


**Figure S2.** **Validation of TNBC-specific miRNAs.** (**A-D**) TTCGA database analysis revealed differential expression of *miR-30a-3p*, *miR-145-3p*, *let-7c-5p*, and *miR-205-5p* between tumor and normal breast tissues. (**E-H**) Kaplan–Meier Plotter analysis showed that high expression levels of *miR-205, miR-145*, *let-7c* and *miR-30a* were associated with improved prognosis in breast cancer patients.


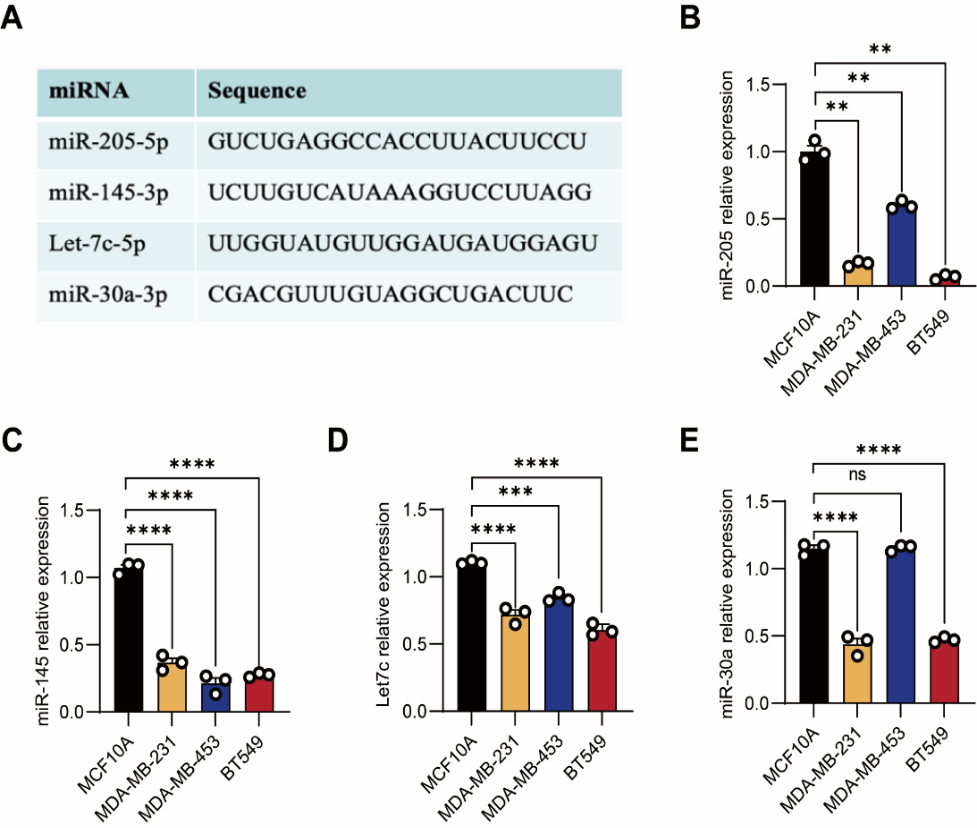


**Figure S3. Cell line-level expression of TNBC-specific miRNAs and target sequences.** (**A**) Sequences of *miR-30a-3p*, *miR-145-3p*, *let-7c-5p*, and *miR-205-5p* were retrieved from the miRBase database. (**B-E**) Expression levels of the four miRNAs were compared between TNBC cell lines (MDA-MB-231, MDA-MB-453, BT549) and normal MCF10A cells.

**
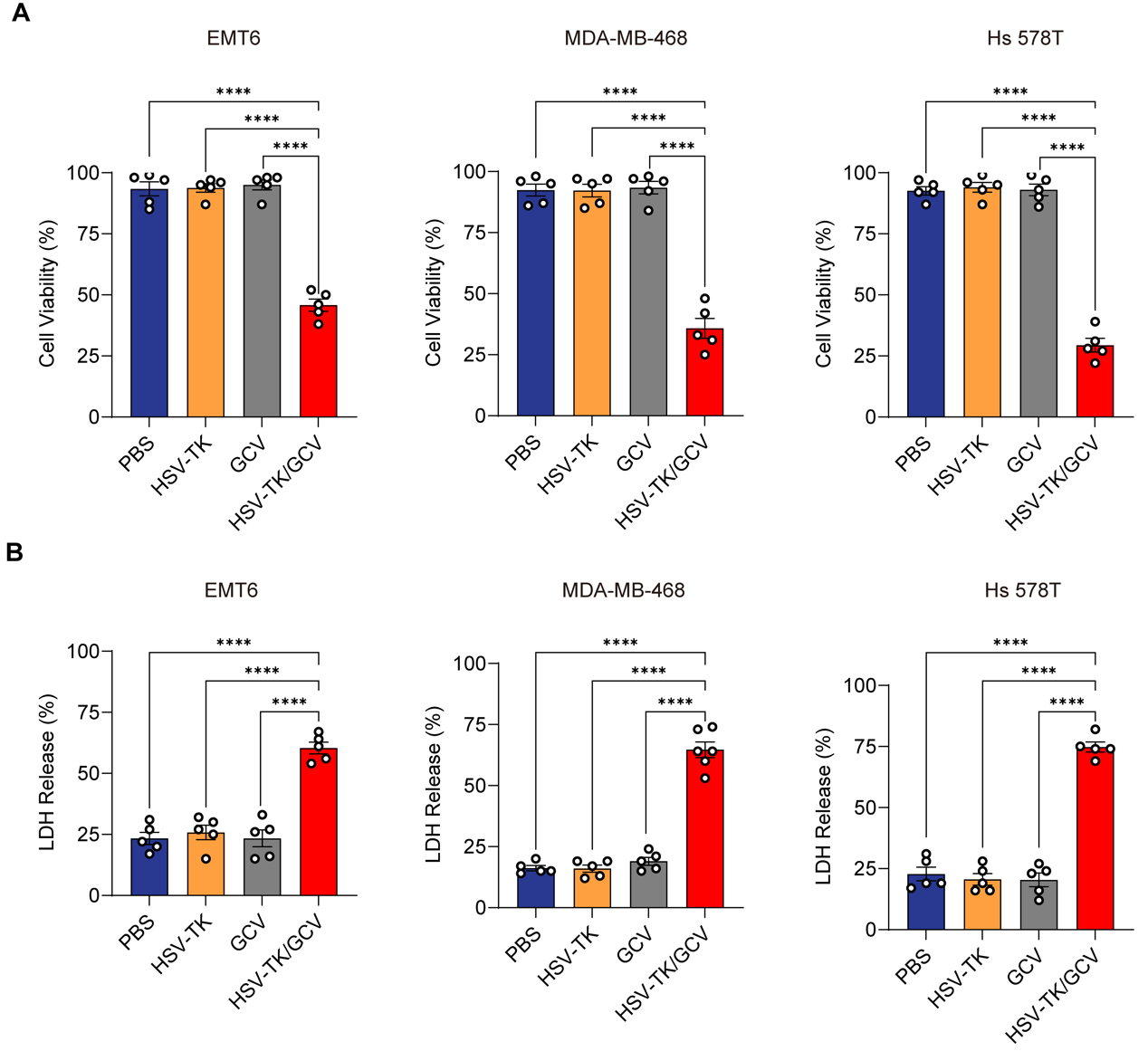
**

**Figure S4. The BRAS circuit for breast cancer-specific expression of therapeutic** **genes HSV-TK in other breast cancer cell lines.** (A, B) Cell viability was measured in BRAS-transduced EMT6, MDA-MB-468 and Hs 578T breast cancer lines after 72 hours using the CCK-8 assay (**A**) and LDH release assay (**B**).

**
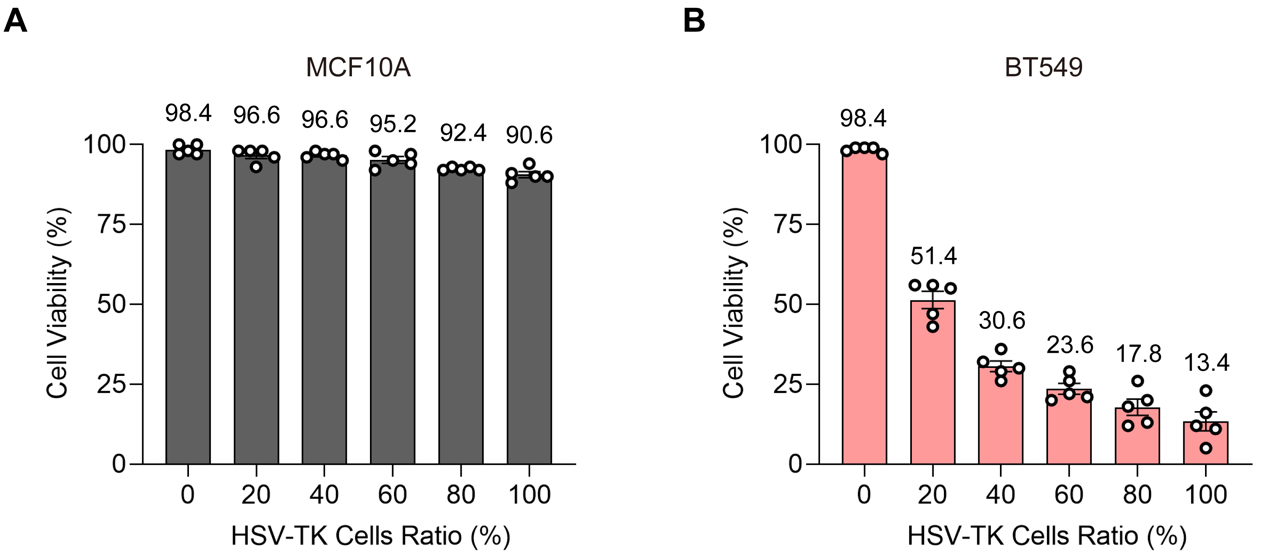
**

**Figure S5. Bystander effect of BRAS circuit mediated therapeutic genes HSV-TK expression.** (A, B) BRAS-transduced BT549 cells were co-cultured with untransduced MCF10A **(A)** or BT549 cells **(B)** in a 96-well plate (1 ×10^4^ total cells per well), in which the percentage of BRAS-transduced BT549 cells is 0%, 20%, 40%, 60%, 80%, and 100%. After 24 h, GCV (3 mg/mL) was added and cytotoxicity was evaluated after 48 hours of GCV exposure by CCK-8 assay. BT549 cells without co-culturing were used as the control to calculate the efficiency of the bystander effect.


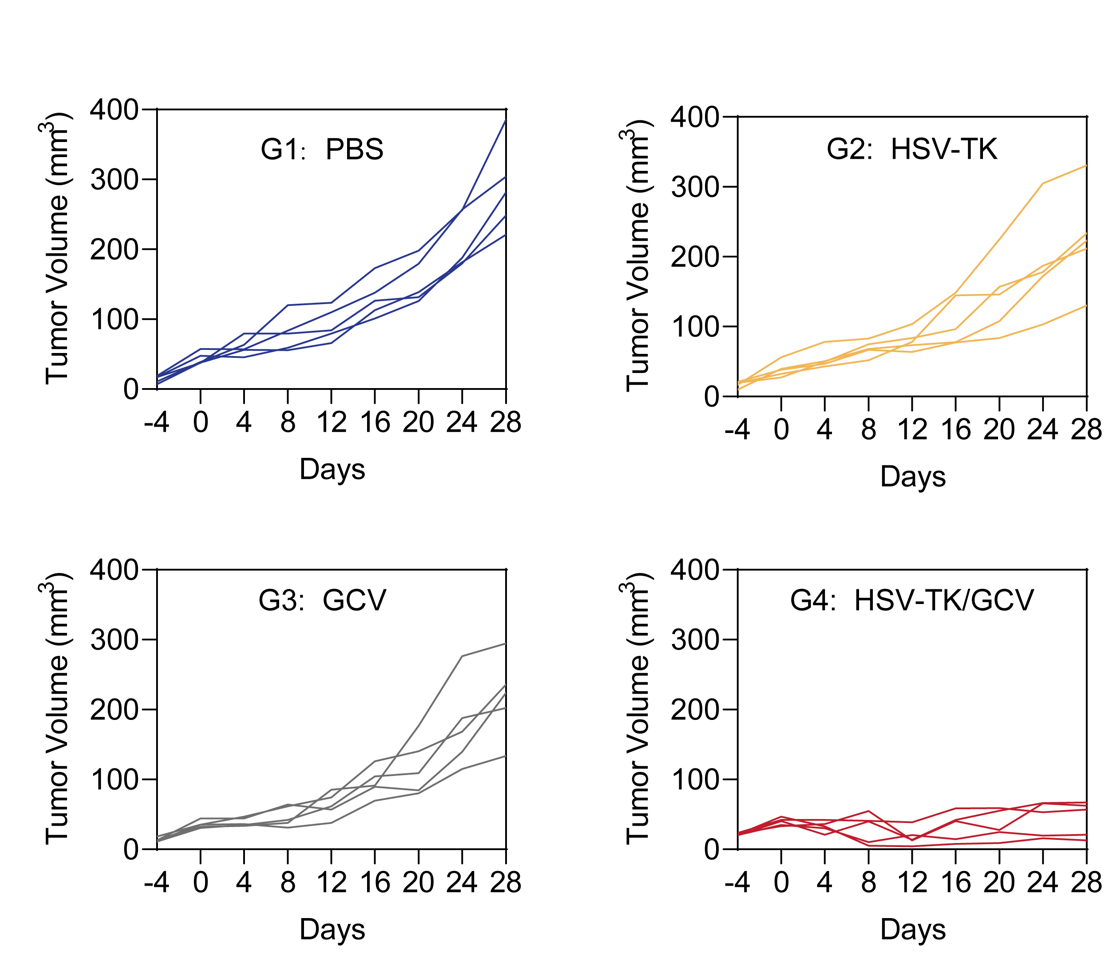


**Figure S6. Individual growth** **curves of BT549 tumor-bearing mice receiving different treatments (related to Fig. 4B).** BT549-bearing mice received intratumoral injections of PBS control(G1), lentiviral vector encoding HSV-TK (G2), GCV alone (G3), and combined BRAS system and HSV-TK/miRNA output (G4). Individual tumor growth curves were recorded every four days until day 28 (*n* = 5).


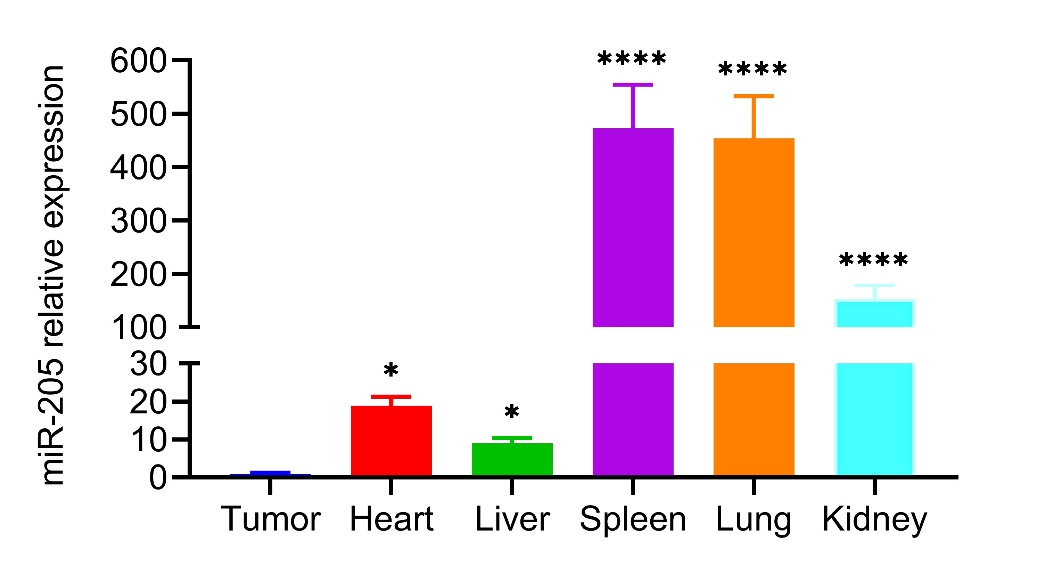


**Figure S7. *miR-205-5p* expression level in the heart, liver, spleen, lung and kidney of mice bearing BT549.** Total RNA was extracted from these tissues, and the expression level of *miR-205-5p* was quantified using qPCR.


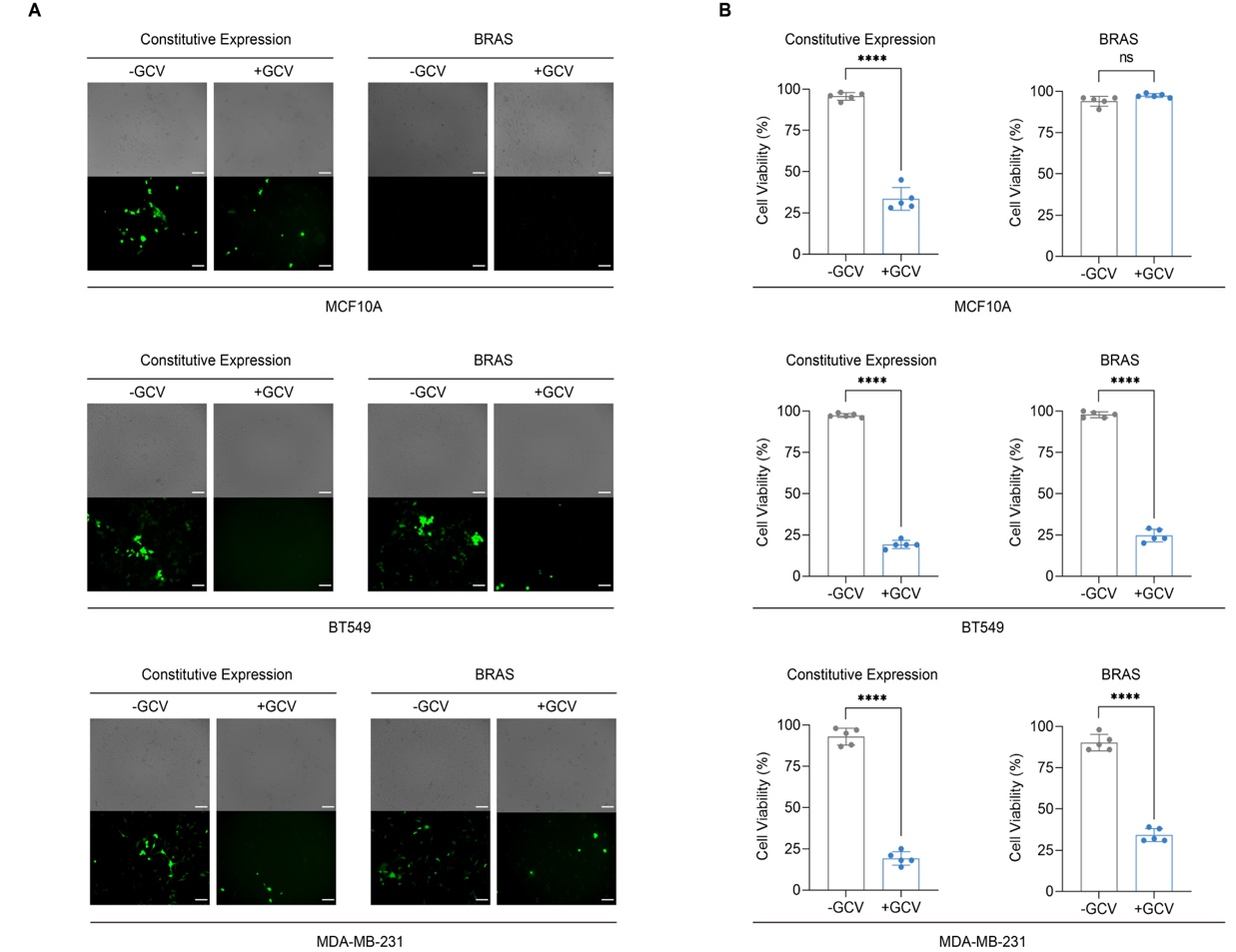


**Figure S8.** Evaluation of off-target toxicity and tumor-killing efficacy between the BRAS and the constitutively expressed HSV-TK/GCV. MDA-MB-231 and BT549 breast cancer lines and normal MCF10A were seeded in 96-well plates at 2,000 cells/well and transduced with lentiviral vector encoding the BRAS circuit: pST171 (LTR-5×UAS-P_hCMVmin_-HSV-TK/GCV-miR205-P2A-EGFP-LTR, MOI = 3) and pST110 (LTR-P_RRM2_-Coh2-Linker-p65HSF1-interval-P_MAFK_-Gal4-Linker-Docs-LTR, MOI = 3) or the constitutive expression pST170 (LTR-P_hCMV_-HSV-TK/GCV-P2A-EGFP-LTR, MOI = 3) in combination with or without 3mg/mL GCV. **(A)** Evaluation of off-target toxicity. EGFP expression was detected using a fluorescence microscope. Scale bar = 100 μm. **(B)** Evaluation of tumor-killing efficacy. Cell viability was measured in BRAS or the constitutively expressed HSV-TK/GCV-transduced MDA-MB-231 breast cancer lines and normal MCF10A cells after 48 hours using the CCK-8 assay.

**Table S1.** **Plasmids designed and used in this study**

| **Plasmid** | **Description and cloning strategy** | **Reference** |
| --- | --- | --- |
| pST10 | Lentiviral vector encoding constitutive P_RRM2_-driven mammalian expression unit for EGFP [LTR-P_RRM2_- EGFP -LTR]. | This study |
| pST11 | Lentiviral vector encoding constitutive P_MAFK_-driven mammalian expression unit for EGFP [LTR-P_MAFK_- EGFP -LTR]. | This study |
| pST15 | Lentiviral vector encoding constitutive P_mamm_-driven mammalian expression unit for EGFP [LTR-P_mamm_- EGFP -LTR]. | This study |
| pST5 | Lentiviral vector encoding constitutive P_MUC1_-driven mammalian expression unit for EGFP [LTR- P_MUC1_- EGFP -LTR]. | This study |
| pST4 | Lentiviral vector encoding constitutive P_PRC1_-driven mammalian expression unit for EGFP [LTR-P_PRC1_- EGFP -LTR]. | This study |
| pST 54 | Lentiviral vector encoding constitutive P_RRM2_-driven mammalian expression unit for Gal4-linker-DocS fusion protein [LTR-P_RRM2_-Gal4-linker-DocS-LTR]. | This study |
| pST55 | Lentiviral vector encoding constitutive P_RRM2_-driven mammalian expression unit for Coh2-linker-p65-HSF1 fusion protein [LTR-P_RRM2_-Coh2-linker-p65-HSF1-LTR]. | This study |
| pST65 | Lentiviral vector encoding constitutive P_MAFK_-driven mammalian expression unit for Gal4-linker-DocS fusion protein [LTR-_PMAFK_-Cal4-linker-Docs-LTR]. | This study |
| pST84 | Lentiviral vector encoding constitutive P_MAFK_-driven mammalian expression unit for Coh2-linker-p65-HSF1 fusion protein [LTR-P_MAFK_-Coh2-linker-p65-HSF1-LTR]. | This study |
| pST81 | Lentiviral vector encoding constitutive P_CMV_-driven mammalian expression unit for Coh2-linker-p65-HSF1 fusion protein [LTR-P_CMV_-Coh2-linker-p65-HSF1-LTR]. | This study |
| pST82 | Lentiviral vector encoding constitutive P_CMV_-driven mammalian expression unit for Gal4-linker-DocS fusion protein [LTR-P_CMV_-Gal4-linker-DocS-LTR] | This study |
| pST83 | Lentiviral vector encoding constitutive 5×UAS-P_hCMVmini_-driven mammalian expression unit for Luciferase and EGFP [LTR-5×UAS-P_hCMVmini_-Luciferase-P2A-EGFP-LTR]. | This study |
| pST107 | Gene circuits output report stable expression vector for Luciferase and EGFP with miRNA205 bind site [LTR-5×UAS-P_hCMVmini_- Luciferase-P2A-EGFP-miRNA205 2BS LTR] | This study |
| pST120 | Gene circuits output report stable expression vector for Luciferase and EGFP with miRNA205 bind site [LTR-5×UAS-P_hCMVmini_-Luciferase-P2A-EGFP-miRNA205 1BS LTR] | This study |
| pST123 | Gene circuits output report Luciferase and EGFP with miRNA145 bind site expression vector [LTR-5×UAS-P_hCMVmini_-Luciferase-P2A-EGFP-miRNA145 2BS LTR] | This study |
| pST124 | Gene circuits output report stable expression vector for Luciferase and EGFP with miRNA145 bind site [LTR-5×UAS-P_hCMVmini_-Luciferase-P2A-EGFP-miRNA145 1BS LTR] | This study |
| pST110 | Lentiviral vector encoding constitutive P_RRM2_-driven mammalian expression unit for Coh2-linker-p65-HSF1 fusion protein and P_MAFK_-driven mammalian expression unit for Gal4-linker-DocS fusion protein [LTR-P_RRM2_-Coh2-linker-p65-HSF1-spacer-P_MAFK_-Gal4-linker-DocS LTR]. | This study |
| pST113 | Lentiviral vector encoding constitutive P_MAFK_-driven mammalian expression unit for Gal4-linker-DocS fusion protein and P_RRM2_-driven mammalian expression unit for Coh2-linker-p65-HSF1 fusion protein [LTR-P_MAFK_-Gal4-linker-DocS-spacer-P_RRM2_-Coh2-linker-p65- HSF1-LTR]. | This study |
| pST122 | Lentiviral vector encoding constitutive P_MAFK_-driven mammalian expression unit for Coh2-linker-p65-HSF1 fusion protein and P_RRM2_-driven mammalian expression unit for Gal4-linker-DocS fusion protein [LTR-P_MAFK_-Coh2-linker-p65-HSF1-spacer-P_RRM2_-Gal4-linker-DocS-LTR]. | This study |
| pST125 | Lentiviral vector encoding constitutive P_RRM2_-driven mammalian expression unit for Gal4-linker-DocS fusion protein and P_MAFK_-driven mammalian expression unit for Coh2-linker-p65-HSF1 fusion protein [LTR-P_RRM2_-Gal4-linker-DocS-spacer- P_MAFK_-Coh2-linker-p65-HSF1-LTR]. | This study |
| pLL3.7 | Lentiviral vector encoding constitutive P_CMV_-driven mammalian expression unit for EGFP LTR-P_CMV_-EGFP-LTR]. | This study |
| pST87 | Lentiviral vector encoding constitutive P_MAFK_-driven mammalian expression unit for Luciferase and EGFP [LTR-P_MAFK_- Luciferase -P2A-EGFP-LTR]. | This study |
| pST88 | Lentiviral vector encoding constitutive P_RRM2_-driven mammalian expression unit for Luciferase and EGFP [LTR-P_RRM2_- Luciferase -P2A-EGFP-LTR]. | This study |
| pST108C | Lentiviral vector encoding constitutive 5×UAS-P_hCMVmini_-driven mammalian expression unit for HSV-TK and Luciferase with miRNA205 binding site [LTR-5×UAS-P_hCMVmini_-HSV-TK-P2A-EGFP-LTR]. | This study |
| pST168 | Lentiviral vector encoding constitutive 5×UAS-P_hCMVmini_-driven mammalian expression unit for HSV-TK and Luciferase with miRNA205 binding site [LTR-5×UAS-P_hCMVmini_-HSV-TK-P2A-EGFP-LTR] and Lentiviral vector encoding constitutive P_RRM2_-driven mammalian expression unit for Coh2-linker-p65-HSF1 fusion protein and P_MAFK_-driven mammalian expression unit for Gal4-linker-DocS fusion protein [ LTR-5×UAS-P_hCMVmini_-HSV-TK-P2A-EGFP-LTR-spacer-LTR-P_RRM2_-Coh2-linker-p65-HSF1-spacer-P_MAFK_-Gal4-linker-DocS LTR]. | This study |
| pST170 | Lentiviral vector encoding constitutive P_hCMV_-driven mammalian expression unit for HSV-TK and EGFP with [LTR -P_hCMV_-HSV-TK-P2A-EGFP-LTR]. | This study |
| pST171 | Lentiviral vector encoding constitutive 5×UAS-P_hCMVmini_-driven mammalian expression unit for HSV-TK and EGFP with miRNA205 binding site [LTR-5×UAS-P_hCMVmini_-HSV-TK-miR205-P2A-EGFP-LTR]. | This study |

**Abbreviations**: **EGFP**, enhanced green fluorescent protein; **Gal4**, galactose-responsive transcription factor 4; **Luciferase**, bioluminescence enzymes; **P2A**, picornavirus-derived self-cleaving peptide engineered for bicistronic gene expression in mammalian cells; **p65**, 65kDa transactivator subunit of NF-kB; **p65-HSF1**, fused transactivator of P65 and heat shock factor 1(HSF1); **pA**, polyadenylation signal; **P_hCMV_**, human cytomegalovirus immediate early promoter; **P_hCMVmin_**, minimal version of P_hCMV_; **UAS**, Gal4-specific binding sequence; DocS/Coh2: an interacting proteins from *Clostridium thermocellu;* **LTR,** Long Terminal Repeat; HSV-TK, Herpes Simplex Virus-Thymidine Kinase;

**Table S2. The primer sequence used in this study**

| Gene name | Forward sequence (5’-3’) | Reverse sequence (5’-3’) |
| --- | --- | --- |
| Hsa_miR-145-3p  (human) | CGCCGGATTCCTGGAAATAC | ATCCAGTGCAGGGTCCGAGG |
|  | RT:GTCGTATCCAGTGCAGGGTCCGAGGTATTCGCACTGGATACGACAGAACA | |
| Hsa_miR-30a-3p  (human) | GCCCTGTAAACATCCTCGAC | ATCCAGTGCAGGGTCCGAGG |
|  | RT:GTCGTATCCAGTGCAGGGTCCGAGGTATTCGCACTGGATACGACCTTCCA | |
| Hsa_miR-205-5p  (human) | GCGTCCTTCATTCCACCGG | ATCCAGTGCAGGGTCCGAGG |
|  | RT:GTCGTATCCAGTGCAGGGTCCGAGGTATTCGCACTGGATACGACCAGACT | |
| Hsa_Let7c-5p  (human) | GCGGCTGAGGTAGTAGGTTGT | ATCCAGTGCAGGGTCCGAGG |
|  | RT:GTCGTATCCAGTGCAGGGTCCGAGGTATTCGCACTGGATACGACACCATA | |
| U6  (human) | GGAGACACGCAAACGGAAG | AGTGCAGGGTCCGAGGTATT |
|  | RT:GTCGTATCCAGTGCAGGGTCCGAGGTATTCGCACTGGATACGACTTGGCG | |
